# Lymphatic pathology in asymptomatic and symptomatic children with *Wuchereria bancrofti* infection in children from Odisha, India and its reversal with DEC and albendazole treatment

**DOI:** 10.1371/journal.pntd.0005631

**Published:** 2017-10-23

**Authors:** Shantanu K. Kar, Bhagirathi Dwibedi, Birendra K. Das, Bikash K. Agrawala, Cherubala P. Ramachandran, John Horton

**Affiliations:** 1 Director of Medical Research, IMS & Sum Hospital, Siksha 'O' Anusandhan University, Bhubaneswar, Odisha, India; 2 Regional Medical Research Centre, Chandrasekhapur, Bhubaneswar, Odisha, India; 3 Utkal Institute of Medical Sciences, Bhubaneswar, Odisha, India; 4 Apollo Hospital, Bhubaneswar, Odisha, India; 5 Universiti Sains Malaysia, Penang, Malaysia; 6 Tropical Projects, Hitchin, United Kingdom; University Hospital Bonn, GERMANY

## Abstract

**Background:**

Once interruption of transmission of lymphatic filariasis is achieved, morbidity prevention and management becomes more important. A study in *Brugia malayi* filariasis from India has shown sub-clinical lymphatic pathology with potential reversibility. We studied a *Wuchereria bancrofti* infected population, the major contributor to LF globally.

**Methods:**

Children aged 5–18 years from Odisha, India were screened for *W*. *bancrofti* infection and disease. 102 infected children, 50 with filarial disease and 52 without symptoms were investigated by lymphoscintigraphy and then randomized to receive a supervised single oral dose of DEC and albendazole which was repeated either annually or semi-annually. The lymphatic pathology was evaluated six monthly for two years.

**Findings:**

Baseline lymphoscintigraphy showed abnormality in lower limb lymphatics in 80% of symptomatic (40/50) and 63·5% (33/52) of asymptomatic children. Progressive improvement in baseline pathology was seen in 70·8, 87·3, 98·6, and 98·6% of cases at 6, 12, 18, and 24 months follow up, while in 4·2, 22·5, 47·9 and 64·8%, pathology reverted to normal. This was independent of age (p = 0·27), symptomatic status (p = 0·57) and semi-annual/bi-annual dosing (p = 0·46). Six of eleven cases showed clinical reduction in lymphedema of legs.

**Interpretation:**

A significant proportion of a young *W*. *bancrofti* infected population exhibited lymphatic pathology which was reversible with annual dosage of DEC and albendazole. This provides evidence for morbidity prevention & treatment of early lymphedema. It can also be used as a tool to improve community compliance during mass drug administration.

**Trial registration:**

ClinicalTrials.gov No CTRI/2013/10/004121

## Author summary

Infection with lymphatic filarial parasites usually occurs early in childhood in endemic areas, but clinical signs appear much later. Reversal of lymphatic pathology has been shown with the much less common *Brugia malayi* infection using DEC and albendazole and there is scarce evidence whether the same occurs with bancroftian filariasis using the above drugs. We designed this study to look for prevalence of lymphatic pathology in children with and without clinical signs of infection and to observe the effect on the pre-treatment pathology using DEC and albendazole given once or twice a year. We have shown, using lymphoscintigraphy, that lymphatic vessel changes occur very early in infection, and treatment can reverse these changes, even when clinical symptoms are already apparent. This has important implications for lymphedema prevention, case management and for advocacy in the Lymphatic Filariasis Elimination Program. It also strengthens the previous evidence of benefit of treatment on early lymphedema management.

## Introduction

Treatment with albendazole and either ivermectin or diethylcarbamazine citrate (DEC) used in the Global Program for Elimination of Lymphatic Filariasis (GPELF) works on the basis of elimination of microfilaria that are infective to the mosquito vector. Transmission within communities is thereby reduced. Based on the data from studies with DEC in India and DEC and ivermectin in the Pacific [1], it has been possible to model the transmission dynamics to estimate the critical levels for parasite survival within populations. The GPELF works on the assumption that parasite prevalence below 1% in any population will lead to failure of effective parasite transmission and eventual extinction within that population. The program targets annual single dose mass drug administration (MDA) for at least five years with population compliance above 80% to achieve this [2, 3].

Providing an effective rationale for individuals, communities, and those countries implementing MDA is a problem, since ‘treating now to prevent in the future’ is a rather tenuous concept, although it is the well-established concept behind all vaccination programmes. It therefore would be helpful to have evidence that intervention is also beneficial to the treated population and particularly to those who are actually infected.

It should also be emphasized that management of chronic filarial disease (lymphedema and hydrocele) is challenging, since currently available morbidity management tools fail to effectively cure advanced disease [4, 5]. A preventive approach would hence be a pre-requisite for morbidity control and one strategy would be to show the beneficial effect of MDA on lymphatic morbidity.

Moore *et al* described DEC induced reversal of early lymphatic dysfunction in a patient with bancroftian filariasis [6]. Clinical trials [7, 8, 9] with long term doxycycline have also demonstrated macrofilaricidal activity and improvement in lymphatic pathology and morbidity (lymphedema and hydrocele) in *W*. *bancrofti* infected populations. Shenoy *et al*.[10, 11] showed that pre-existing lymphatic pathology caused by *Brugia malayi* could be reversed with single dose DEC and albendazole. Using lymphoscintigraphy to visualize the lymphatics and the collateral circulation that developed during symptomatic and asymptomatic infection, they demonstrated a significant and sustained improvement in the lymphatic circulation following treatment. Although the available literature gives indication that lymphatic pathology can also be reversible in *W*. *bancrofti* filarial disease, long treatment with doxycycline may not have wide acceptance and the limited results from a *B*. *malayi* population would not be directly applicable. Furthermore, *W*. *bancrofti* and *B*. *malayi* are not only different in terms of transmission and disease development, adult worms responsible for lymphatic morbidity are also different in size and localization. Hence, it was felt essential to generate more evidence on clinical or sub clinical lymphatic pathology and its reversibility in the geographically more wide spread parasite, *W*. *bancrofti*, causing lymphatic filariasis (LF), using the drugs used in the elimination program.

Bal *et al*.[12] clearly showed that *W*. *bancrofti* infection, as demonstrated by circulating filarial antigen (CFA), is mostly acquired at an early age (3-5yrs) in an endemic area; while microfilaraemia develops later and progressively increases over time. These data suggest that intervention needs to occur as early as possible.

Even if microfilaria clearance is the proven impact of MDA, there is no current evidence that MDA is of benefit to the young asymptomatic population with respect to bancroftian filarial disease. Thus, the asymptomatic period after initial infection in early childhood until development of lymphedema and hydrocele leaves a window to be explored.

The present study was designed to (a) determine the prevalence of lymphatic pathology in children with *W*. *bancrofti* infection aged between 5 and 18 years with or without symptoms of filarial disease using lymphoscintigraphy and (b) assess any improvement or reversal of the existing lymphatic pathology, using repeated lymphoscintigraphy, after treatment with annual or bi-annual doses of DEC and albendazole as used in the current MDA program in India.

## Methods

**Fig 1:**
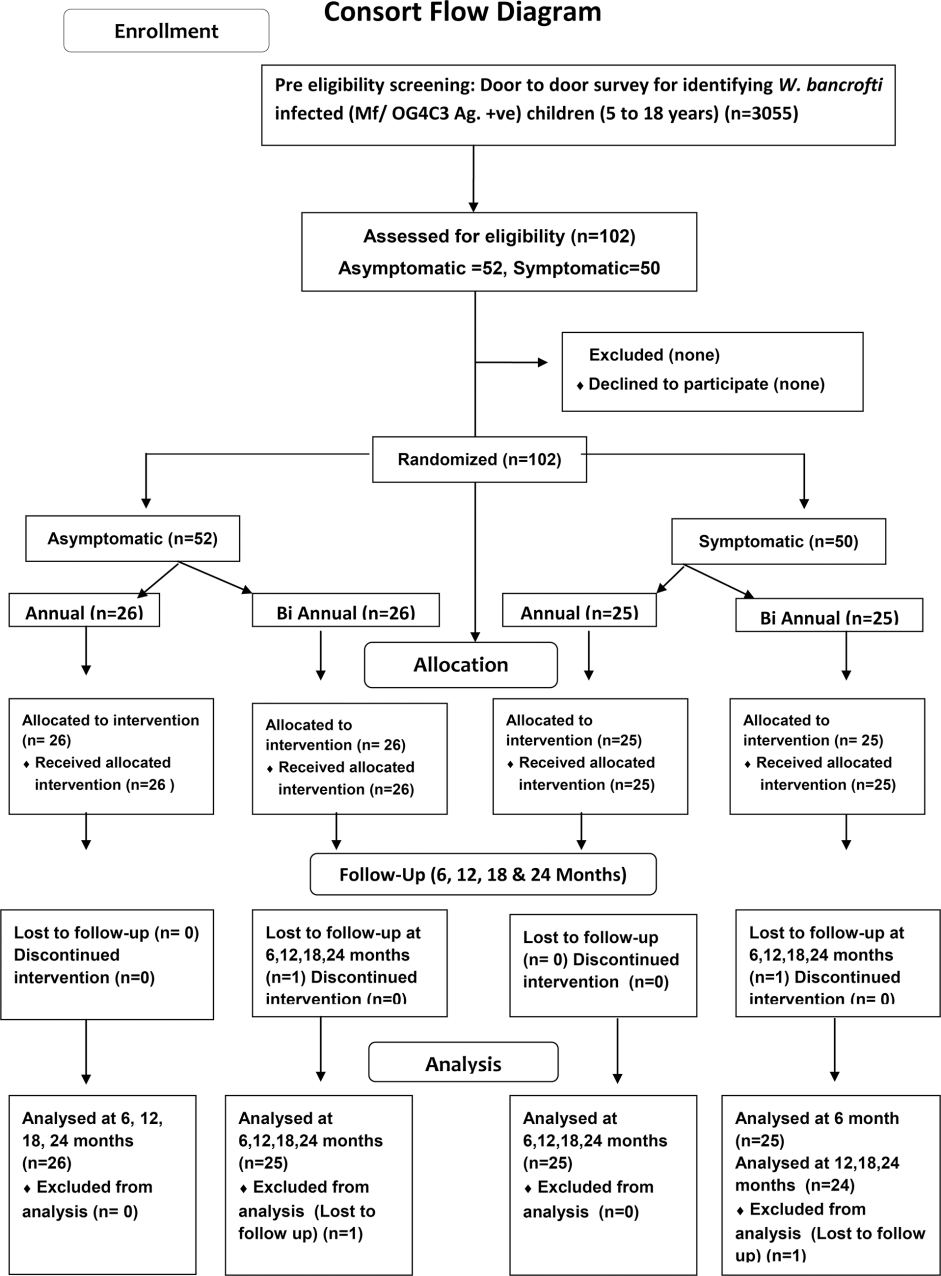
Consort flow diagram.

Subjects with *W*. *bancrofti* infection identified by community level screening were evaluated at baseline for evidence of lymphatic pathology and prospectively followed as an observational cohort with drug intervention i.e. DEC and albendazole as in the MDA program, while assessing the effect on the baseline lymphatic abnormality (Fig 1). The study was conducted between October 2009 and April 2014 which covered the selection of endemic villages, screening, enrolment and follow up of the study subjects for two years after baseline evaluation. The study outline is reflected in Fig 1.

### Study area and population

The study enrolled eligible participants from a *W*. *bancrofti* endemic area of Khordha district in Odisha State, India. Twelve villages with microfilaria positivity of ≥ 5% detected by rapid screening were selected for the study. All were located between 20.17° to 20.27° N latitude and 85.67° to 85.84° E longitude within 15–50 kilometres of the Regional Medical Research Centre (Indian Council of Medical Research) in Bhubaneswar.

Assuming a prevalence of lymphatic pathology in 70% of *W*. *bancrofti* infected children, based on the findings from Shenoy et al [10,11], the sample size was calculated as 100 subjects, at 90% confidence level with 0.15 width of confidence interval. This sample would also be sufficient if 80% of those showing lymphatic abnormality would show improvement after treatment. Hence, it was targeted to include around 100 children in the target age group (5 to 18 years) with *W*. *bancrofti* infection.

### Inclusion and exclusion criteria

Children between 5 and 18 years of age with evidence of *W*. *bancrofti* infection with adult worm (Og4C3) antigenemia with/without microfilaraemia were considered eligible for enrolment into the study. Pregnant females were excluded from the study. Subjects with serum ALT ≥ 30 IU/l, creatinine ≥ 1.2 mg/dl, hemoglobin level < 10gm/dl, or evidence of other systemic illnesses were also excluded from enrolment.

### Eligibility screening and subject enrolment

Baseline screening was done in night camps arranged in the villages, after initial awareness generation activities had been carried out by the study team. Prior to enrolment, written consent was obtained from parents or guardians in case of minors and from individuals who were 18 years of age. History of filarial disease manifestations were recorded in a pre-designed format and included present or past history of adeno-lymphangitis, chyluria, recurrent hematuria, lymphedema or hydrocele, and any antifilarial treatment received by the individual. Clinical examination was undertaken to detect any other existing systemic illness and for signs of filarial disease, particularly adeno-lymphangitis, lymphedema and hydrocele and recording the grade of lymphedema [13]. Enrolment was continued until 100 eligible children (50 symptomatic and 50 asymptomatic) had been identified.

### Sample collection and lab investigation

Night blood samples (4–5 ml) were collected aseptically between 21:00 and 23:00 hours. The sample was divided into 2 aliquots: first aliquot of 2ml blood preserved in EDTA and the second aliquot stored in plain vial for separation of serum. The serum samples were stored at -20°C and the EDTA collected sample was preserved at 4°C.

Microfilaria counts in the individual specimens were determined by microscopic examination of 1ml EDTA preserved blood concentrated by Nuclepore membrane filtration technique. Adult worm antigen (Og4C3) was measured from serum using the TropBio ELISA test kit (TropBio, Townsville, Australia), following the manufacturer’s instructions [12].

Serum ALT and creatinine were estimated by an automated biochemistry analyser (Cobas Integra 400, Roche). Blood cell counts and hemoglobin estimation were done in a cell counter (MS-4, Melet Schloesing Laboratories, France). Pregnancy in females between 14–18 years of age was ruled out by a urine β-hCG test.

### Pre-treatment ultrasonography & lymphoscintigraphy of enrolled children

After eligibility screening, all subjects were investigated by Doppler ultrasonography using a 7 to 12 MHz probe (GE Logique 400 PRO, Wipro) to detect the filarial dance sign (FDS) of adult filarial worms in axillary, inguinal and scrotal areas.

To document lymphatic abnormality in the lower limbs, TC^99^ labelled radio-nucleotide lymphoscintigraphy was used as described below.

#### Lymphoscintigraphy:

Lymphoscintigraphy (LSG), the radionuclide technique for imaging the lymphatic system using interstitially injected colloidal particles is considered safe and the gold standard for assessing lymphatic flow [14, 15].

In this study Tc^99^ labelled sulphur colloids (2 millicurie) were injected intra-dermally in the webs between the first and second toes of each foot. Images (GE Millenium Dual Head Gamma Camera) of the feet, lower limbs (knee and thigh regions) and pelvis were taken at 0, 10, 30 and 60 minutes post injection and a whole body survey taken at two hours. Radiation exposure was negligible since only a small part of the colloidal particles enter into the systemic circulation.

All evaluations of lymphatic flow and analysis were undertaken on stored images by one of the authors (BKD) at the Utkal Institute of Medical Sciences, Bhubaneswar, Odisha, India. Individual subjects acted as their own controls, with changes evaluated from the baseline pre-treatment situation. Lymphatic flow from foot level to inguinal region was interpreted by comparing the visual images of tracer uptake at foot, popliteal and inguinal levels over time. In addition, a semi quantitative assessment was done by calculating the percentage of tracer accumulating in inguinal lymph nodes in comparison to the site of injection at different times after subtracting the proportion of tracer in the region of interest between the two points.

To document lymphatic abnormality the following diagnostic points were used in this study [10, 16]. The first and most important was whether there was increased accumulation of radio tracer in lymphatic webs and soft tissue. This pattern not only indicates the existence of lymphostasis but also the range and severity of the disease. The second was whether inguinal-iliac and para-aortic nodes could be seen on the image. None or poor visualization of these lymph nodes indicates a more severe state of the disease with reduced centripetal flow. The third was whether there were abnormal lymph nodes or lymphatic branches visible in the extremities. All scans were evaluated in both limbs looking for following criteria:

1. Persistence of lymphatic channel visualization in the lower limbs.
2. Existence of multiple collateral lymphatic channels.
3. Persistent visualization of popliteal lymph nodes.
4. Poor or non-visualization of inguino-iliac and para-aortic lymph node chains.
5. Incremental percentage of tracer concentration in inguino-iliac region in images taken at different intervals.

**Fig 2:**
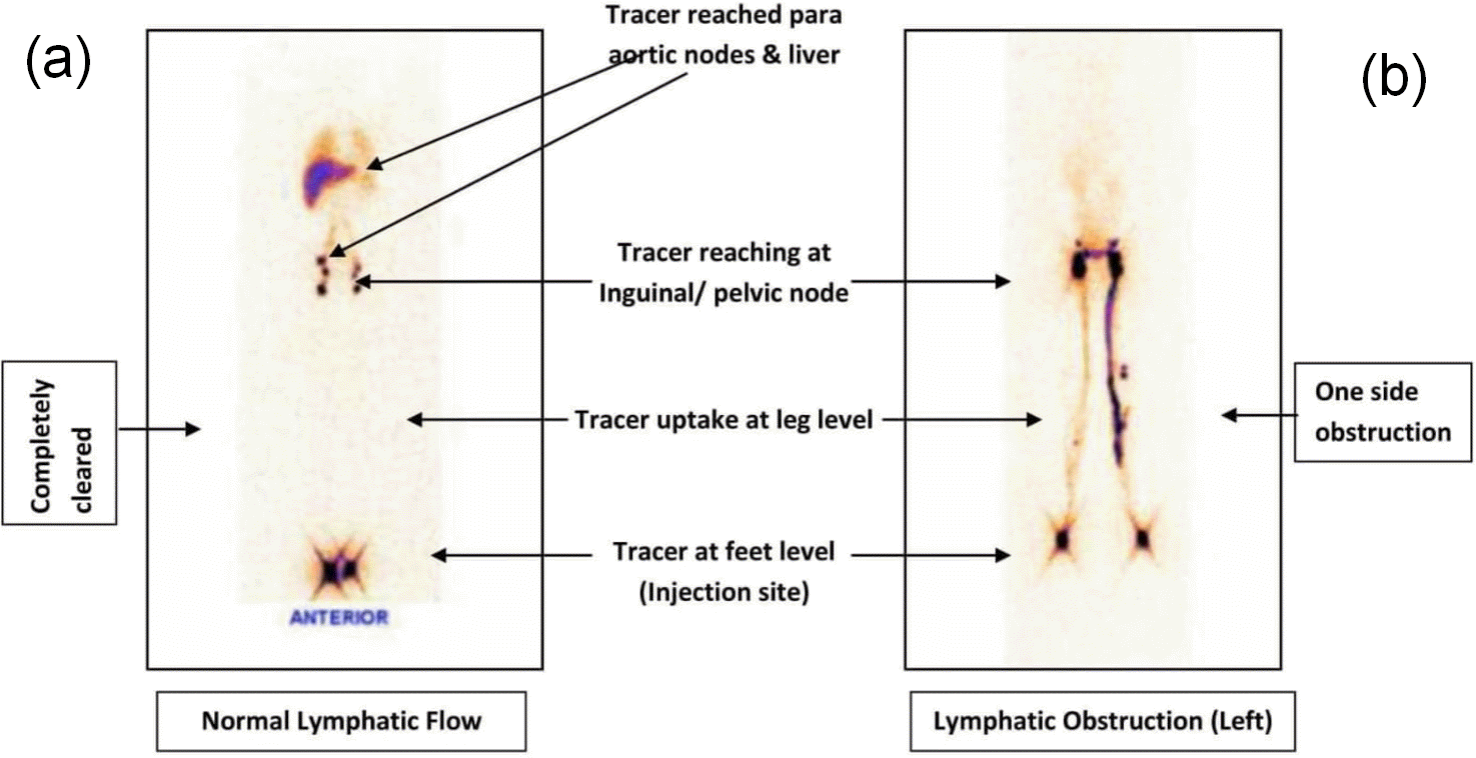
Representative image of whole body lymphatic scan of (a) normal lymphatic flow and (b) lymphatic flow obstruction. These images are given for better understanding of the lymphoscintigraphy imaging made in the study. Subsequent figures (Figs 3–10) are provided as cross sectional images at feet, popliteal and inguino-pelvic label taken at different time points to reflect the change over time.

Images of normal lymphatics and lymphatic pathology/abnormality are shown in Fig 2. Repeat LSG was visually compared to the pre-treatment images to determine whether any change of lymphatic flow or pattern had occurred over time after drug intervention.

### Randomization after enrolment

The study was conducted as a parallel group randomized study. It was planned to enrol 100 subjects with *W*. *bancrofti* infection, half being asymptomatic, and half with some evidence of filarial disease. Subjects in both asymptomatic (n = 52) and symptomatic (n = 50) groups were randomized, using a list generated using Prism software (Graph Pad Prism 6.0), into equal subgroups to receive either six monthly (semi-annual) or yearly (annual) treatment.

### Supervised drug dosing and adverse event (AE) monitoring

On completing baseline investigations, all subjects were administered a single oral dose of diethylcarbamazine citrate (DEC) tablets (Banocide forte, GSK, Nasik, India) and albendazole tablets (Zentel, GSK, Solan, India) supervised by the investigators. The albendazole dose was 400mg for all ages while the DEC dose was 200mg for 5 to 14 years and 300mg for 15 to 18 years of age, as recommended by the National Filariasis Elimination Program, NVBDCP, India [17]. Drugs were taken at home, supervised by the study personnel. Follow up household visits were made to record and manage any adverse events, daily for a week or until resolution of any events following drug administration.

### Follow up of the subjects

Study participants were enrolled in batches of four to six, treated according to the predetermined randomization list and followed at six monthly intervals with drug dosing as appropriate and repeat investigations. After baseline evaluation, all children received the first dose of DEC and albendazole in doses appropriate to their age. DEC and albendazole treatment was repeated as follows. The semi-annual group received treatment at 0, 6, 12, 18 and 24 months and the annual group received treatment at 0, 12 and 24 months. Hematological investigations, urine pregnancy test, and lymphoscintigraphy were repeated in each of the participants at each follow up point, while ultrasonography was repeated only on subjects who had demonstrated FDS at baseline.

One hundred children completed successful follow up at the different time points and were available for analysis. One symptomatic child was withdrawn shortly after baseline evaluation with a diagnosis of pulmonary tuberculosis and one asymptomatic subject was lost to follow up from 12 months onwards. Both were in the semi-annual treatment group.

### Data analysis

Individual information from baseline screening to last follow up visit at 24 months were recorded in a pre-designed form and entered into an Excel database. Data accuracy was ensured by double data entry, matching and subsequent data cleaning. Data analysis was performed using SPSS version 16 with appropriate tools for parametric and non-parametric variables. The results were expressed as percentage frequency and percentage changes. Chi-square test was used to compare the proportions.

### Ethics statement

The study was undertaken following ICH-GCP guidelines with written informed consent from parents, guardians, or individuals as applicable. The protocol and consent information was approved by Human Ethical Committee of Regional Medical Research Centre, Bhubaneswar. The study protocol was further reviewed by the Indian Council of Medical Research (ICMR) and Health Ministry Screening Committee, Govt. of India. Patient safety was assured by monitoring and management of adverse events. The study was registered under CTRI, ICMR (No CTRI/2013/10/004121).

## Results

The study population was mostly of middle and lower socio- economic status living in an agriculture-based economy. In total, 3055 children between 5 and 18 years of age were screened to identify *W*. *bancrofti* infected subjects who were either Og4C3 antigen positive (n = 480, 15·5%) or Mf positive (n = 154, 5%). From them, 100 individuals were screened for eligibility after obtaining written informed consent. All of them satisfied the inclusion and exclusion criteria and were enrolled into the study. Fifty had signs or symptoms of filarial disease and are referred to as ‘symptomatic’ while the remaining 50 without signs and symptoms of disease are referred to as ‘asymptomatic’. Due to early drop-out of two subjects, these were replaced, both in the asymptomatic group. Only data from those who completed the 24 months of follow-up were analysed. The age and sex distribution of the children is presented in Table 1. There were more males than females in all groups. It was originally intended that there would be a greater proportion of younger (pre-pubertal) children in the population, although this was not a primary criterion for enrolment. In the event, significantly more symptomatic subjects (74%) were found in the older (12–18 years) age group (p = 0.02), leading to a greater proportion of older children overall.

**Table 1:** Age& gender distribution of enrolled subjects.

Age (years)	Asymptomatic		Symptomatic		Total
	Male	Female	Male	Female	
5–11	15	10	8	5	38
12–18	21	6	27	10	64
**Total**	**36**	**16**	**35**	**15**	**102**

A history of adeno-lymphangitis (n = 32), early grade (I or II) lymphedema (n = 11), hydrocele (n = 6) and microscopic hematuria (n = 1) were the filarial disease manifestations recorded in the symptomatic individuals.

Thirty (29·5%) of the enrolled subjects were microfilaraemic with counts ranging from 30 to 1540/ml of blood. All the subjects (n = 102) had circulating filarial antigen (Og4C3), the antigen level ranging from 182 to 15107 units.

Ultrasonography detected filarial dance sign (FDS) of adult worms in the inguino-scrotal area in 9 (8.8%) of the enrolled subjects. Lymphoscintigraphy studies at baseline revealed lymphatic abnormality/ pathology in 73 (71·6%) cases (Table 2).

**Table 2:** Status of *W*. *bancrofti* infection and prevalence of lymphatic pathology/abnormality at baseline.

	Asymptomatic (n = 52)	Symptomatic (n = 50)	Total (n = 102)	P value
Number of subjects with microfilaraemia (%)	20 (38.5)	10 (20.0)	30 (29.4)	**0.033**
Number of subjects with circulating filarial antigen (OG4C3) (%)	52 (100)	50 (100)	102 (100)	**—**
Number shown filarial dance sign of adult worm in ultrasonography (%)	4 (7.7)	5 (10.0)	9 (8.8)	**0.475**
Number shown lymphatic pathology/ abnormality in lymphoscintigraphy (%)	33 (63.5)	40 (80.0)	73 (71.6)	**0.051**

**Fig 3:**
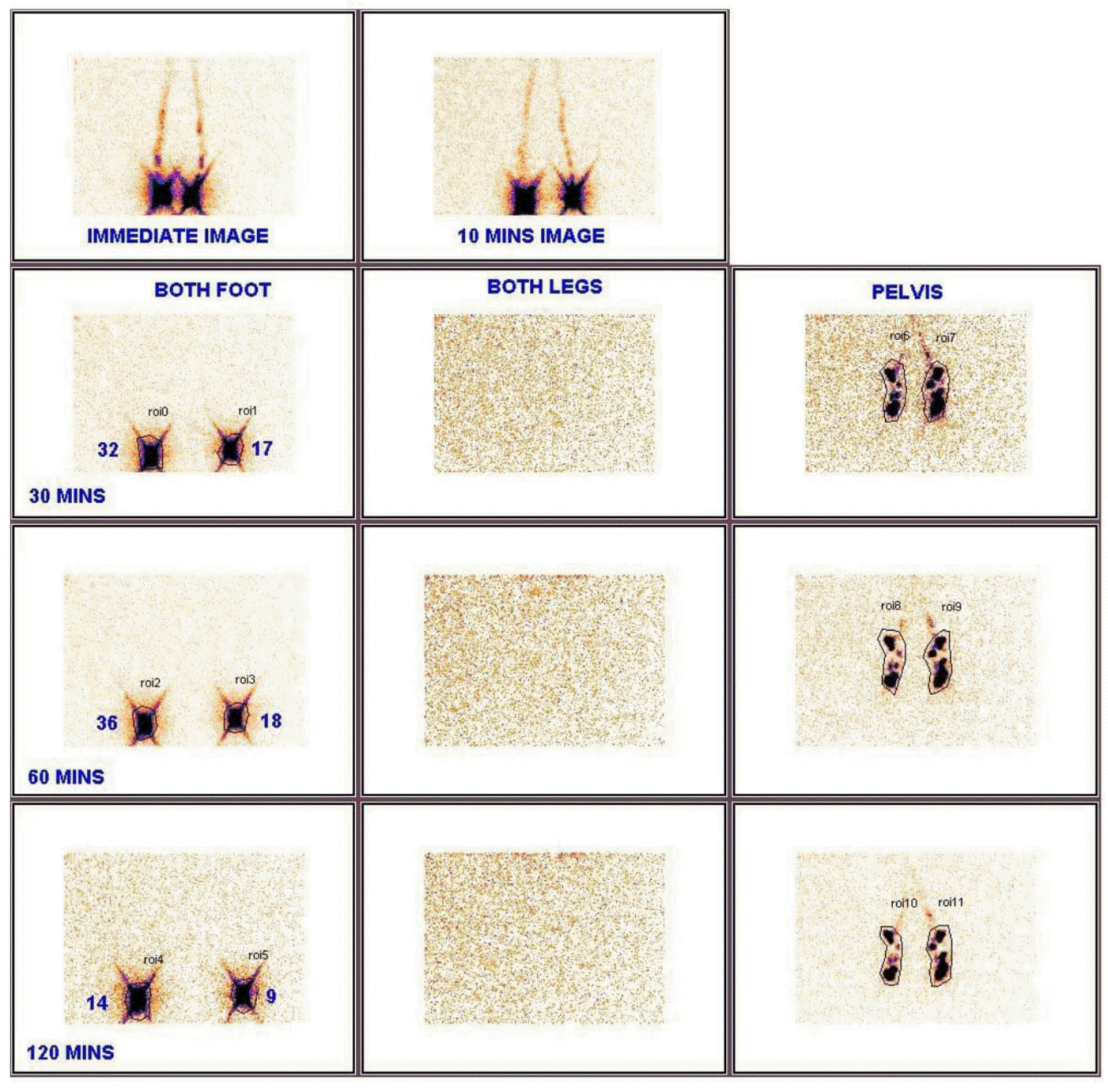
Normal images: 10, 30, 60, & 120 minute images of lymphoscintigraphy in a 14 yr male (asymptomatic and Mf positive). In both the limbs, at 10 minutes tracer movement into the lymphatics is seen in both legs and at 30 minutes inguinal nodes are visualized, lymphatic tract not imaged and tracer uptake symmetrical hence lymphatic flow normal in both legs. Note the granular appearance in leg images is typical of background noise where there is no additional tracer image.

**Fig 4:**
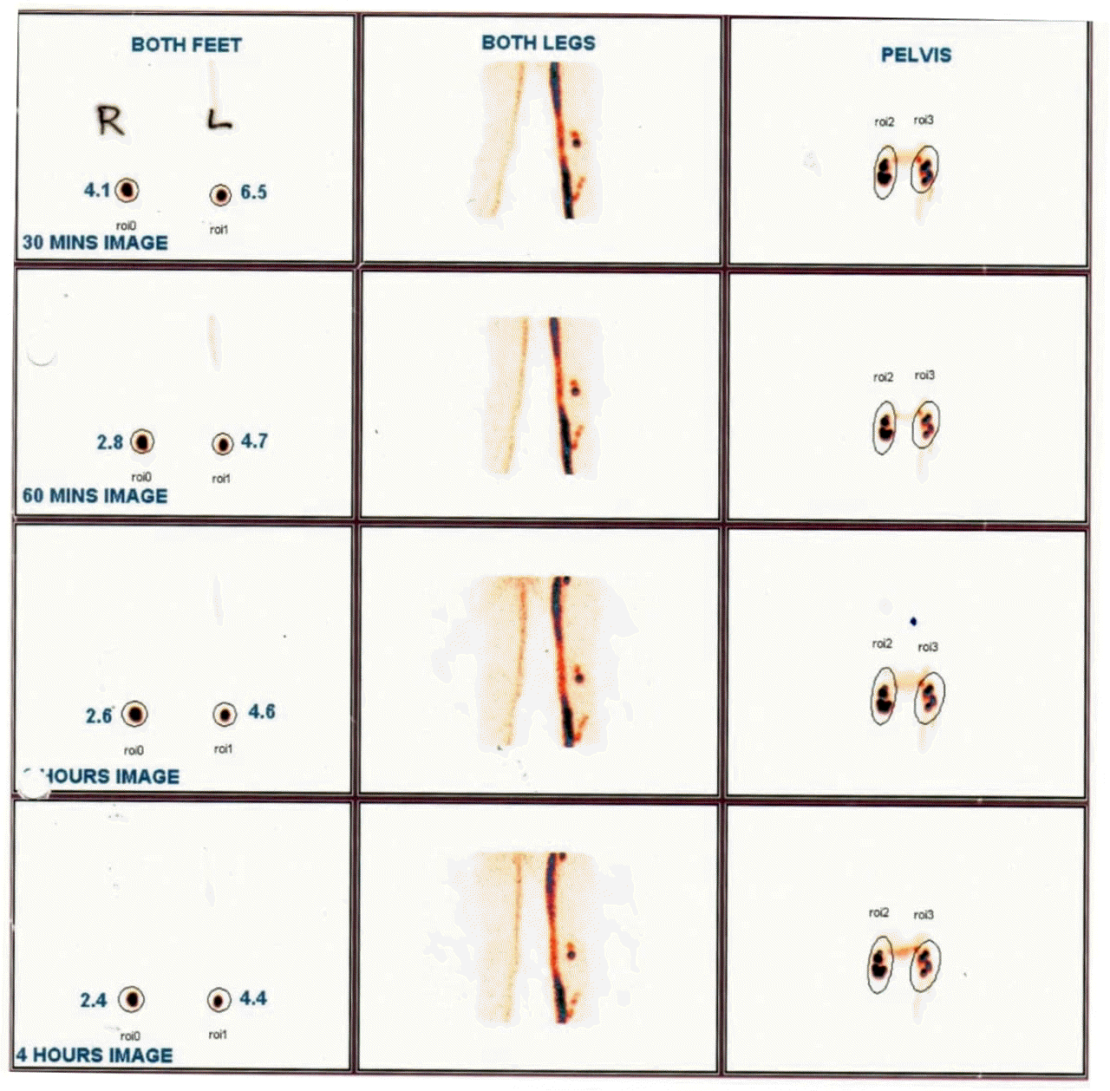
Baseline 30, 60 & 120 minute images showing lymphatic pathology in left leg of a 12 year old male (symptomatic & Mf positive). In the left leg, the lymphatic channel well visualized, and tracer uptake at injection site is low indicating lymphatic flow obstruction in left leg.

**Fig 5:**
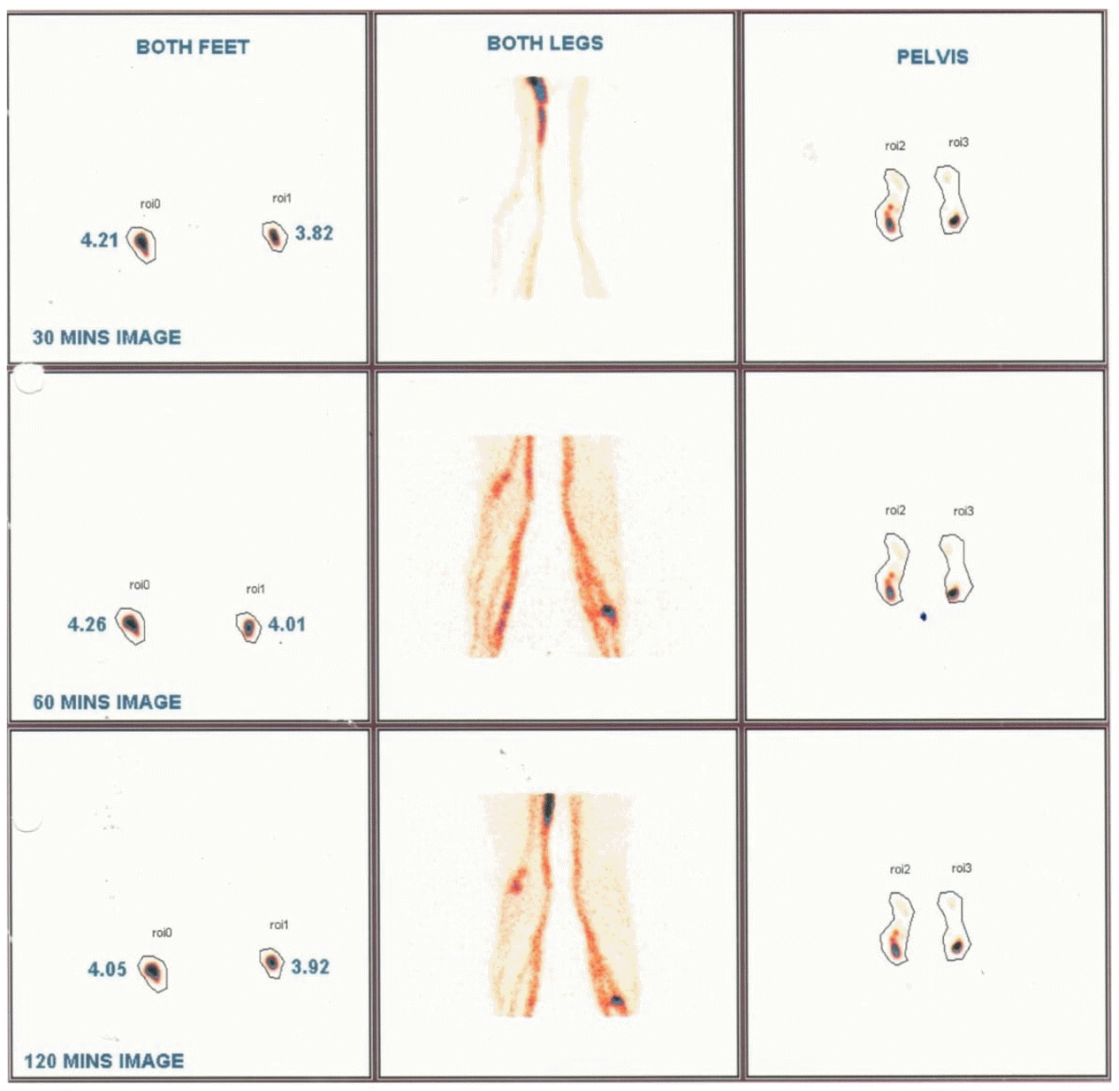
Baseline 30, 60 & 120 minute images showing lymphatic pathology in left leg of a 11 year old male (symptomatic & Mf positive). In right leg, the lymphatic channel well visualized, and tracer uptake at injection site is low indicating lymphatic flow obstruction in left leg. There is also some delay in uptake in the left leg, and both legs have evidence of lateral channel formation.

**Fig 6:**
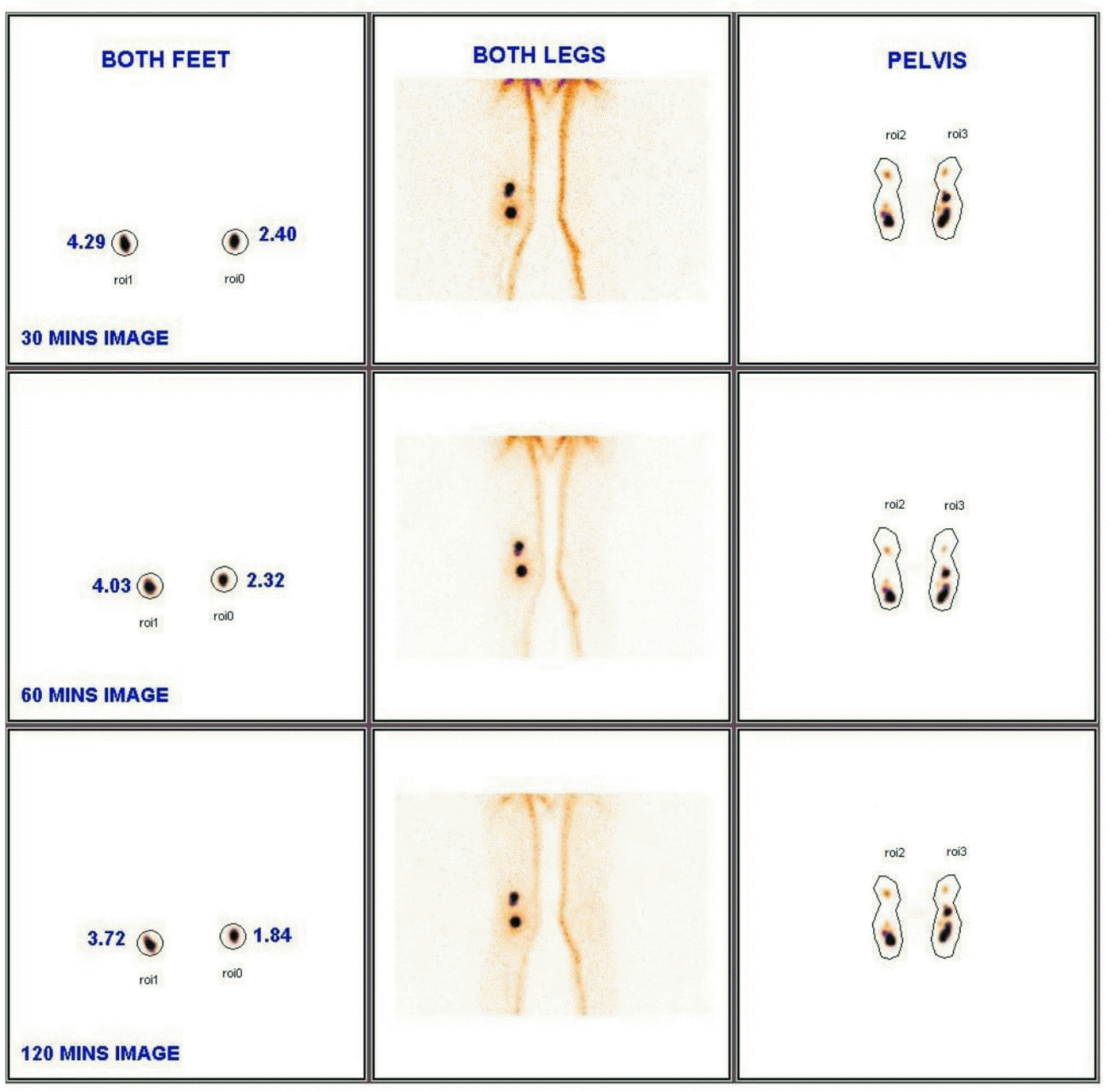
Baseline 30, 60, & 120 minute images of lymphoscintigraphy in a 8 year old male (asymptomatic and Mf positive). Images show visualization of popliteal nodes and fewer inguinal nodes on right leg indicating tracer accumulation at popliteal level hence lymphatic flow obstruction.There is also delayed clearance from the injection site on the right foot.

At baseline, LSG demonstrated sluggishness in lymphatic flow in one of the lower limbs in 60 (82%) cases of the 73 children with abnormalities. Collateral lymphatic channels were visualized in 9 (12·3%) and persistent visualization of popliteal lymph nodes was recorded in 25 (34·2%) of the children with lymphatic pathology. The youngest child with lymphatic flow abnormality was an asymptomatic 6 year old male with circulating filarial antigen and no microfilaraemia. Examples of abnormalities of lymphatic flow in children prior to treatment are shown in Figs 3–6.

The post treatment evaluation of LSG images with the baseline lymphatic pathology/abnormality showed improvement in 70·8, 87·3, 98·6 and 98·6% of children at 6, 12, 18 and 24 months respectively (Table 3). Moreover a return to a normal LSG image was seen in 4·2, 22·5, 47·9 and 64·8% of cases at the same time points post treatment.

**Table 3:** Changes in lymphatic pathology/ abnormality in asymptomatic & symptomatic children following drug intervention.

Period	Improvement in Lymphatic pathology/ abnormality (%)			Complete reversal of Lymphatic pathology/ abnormality (%)		
	Asymptomatic	Symptomatic	Total	Asymptomatic	Symptomatic	Total
6 months	23/32 (71.9)	28/40 (70.0)	51/72 (70.8)	2/32 (6.2)	1/40 (2.5)	3/72 (4.2)
12 months	29/32 (90.6)	33/39 (84.6)	62/71 (87.3)	7/32 (21.9)	9/39 (23.1)	16/71 (22.5)
18 months	31/32 (96.9)	39/39 (100)	70/71 (98.6)	14/32 (43.8)	20/39 (51.3)	34/71 (47.9)
24 months	32/32 (100)	38/39 (97.4)	70/71 (98.6)	20/32 (62.5)	26/39 (66.7)	46/71 (64.8)

**Fig 7:**
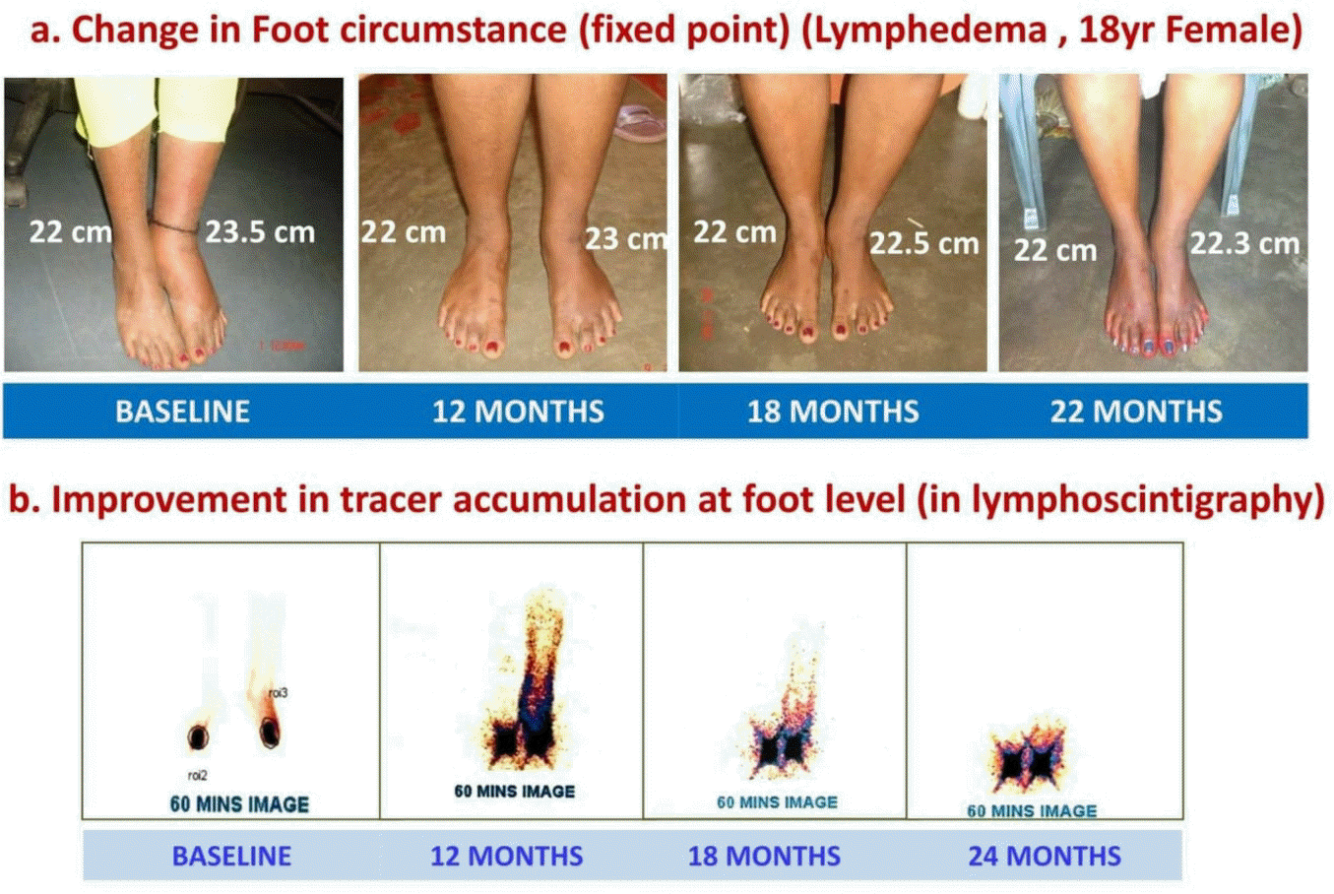
a & b: Post treatment reversibility of lymphedema of left leg in a symptomatic 18 year old female. (a) Change in foot circumference (fixed point over lateral maleolus). (b) Improvement in tracer accumulation at foot level (in lymphoscintigraphy).

**Fig 8:**
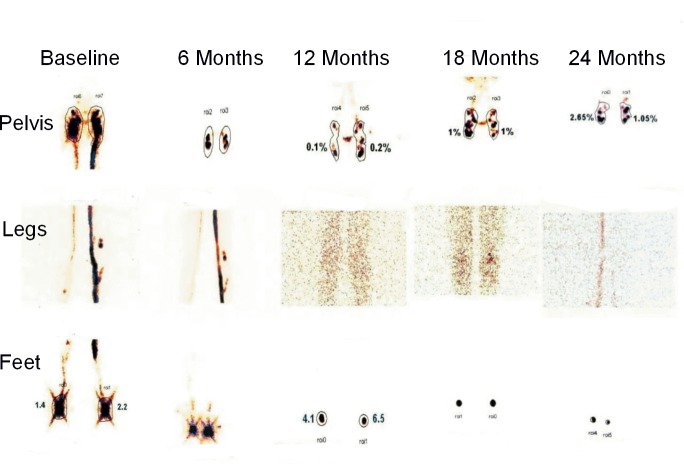
Post treatment reversibility in a 12 year old asymptomatic male. Images show lymphatic flow obstruction on left leg at baseline, with complete reversal apparent at 1 year & sustained at 18 & 24 Months.

**Fig 9:**
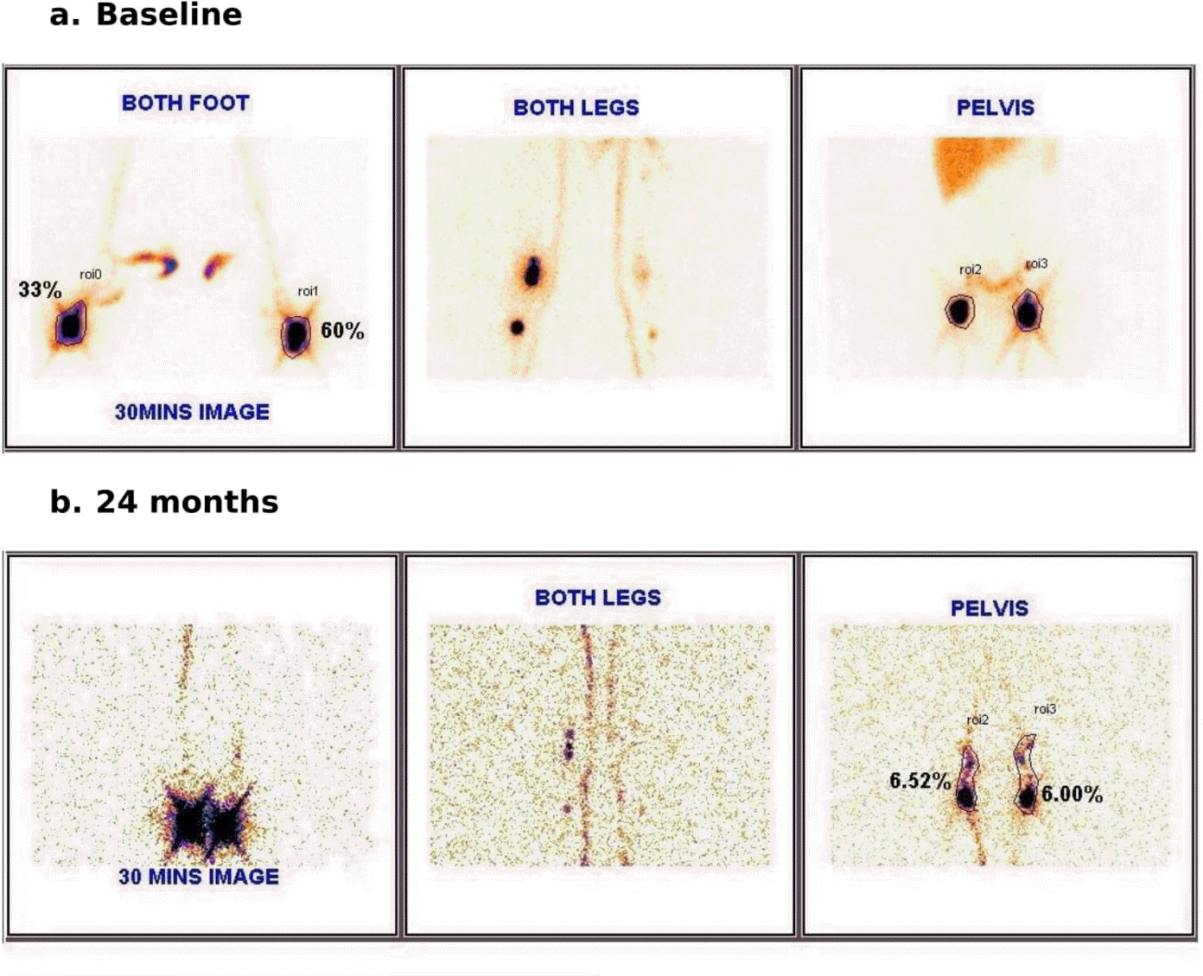
a & b: Baseline and 24 month images showing disappearance of tracer retention in popliteal lymph nodes. Lymphatic flow in both legs is improved (a) baseline. (b) 24 months.

**Fig 10:**
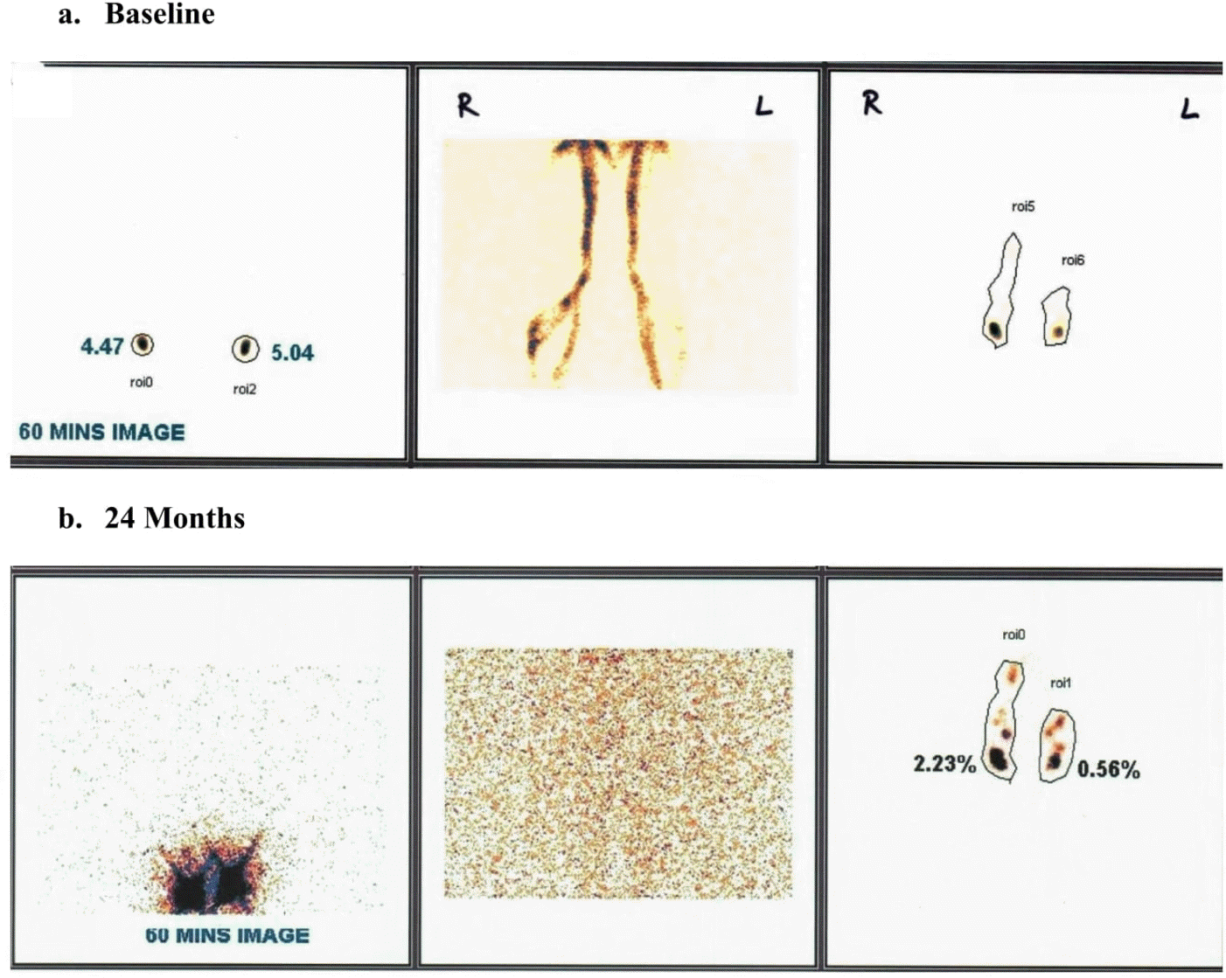
a & b: Baseline and 24 month images showing disappearance of tracer retention in collateral lymphatic channels and overall improvement in lymphatic flow. Leg images are normal at 24 months. (a) baseline. (b) 24 months.

Examples of improvement/normalization in lymphatic flow, disappearance of collateral channels and popliteal nodes following treatment are presented in Figs 7–10.

There was no statistically significant difference in the frequency of improvement or reversal of the baseline lymphatic abnormality/pathology in comparisons between symptomatic and asymptomatic subjects (p = 0·57) (Table 3) or between annual and semi-annual dose groups (p = 0·46).

At 12 months follow up higher percentage of improvement was observed in mf positive subjects (p = 0.02) (Table 4) and males (p = 0.009) (Table 5).

**Table 4:** Changes in lymphatic pathology/ abnormality in children who were positive and negative for microfilaria at baseline.

Period	Improvement in Lymphatic pathology/ abnormality (%)			Complete reversal of Lymphatic pathology/ abnormality (%)		
	MF +ve	MF -ve	Total	MF +ve	MF -ve	Total
6 months	15/19 (78.9)	36/53 (67.9)	51/72 (70.8)	1/19 (5.3)	2/53 (3.8)	3/72 (4.2)
12 months	18/19 (94.7)	44/52 (84.6)	62/71 (87.3)*^a^	8/19 (42.1)	8/52 (15.4)	16/71 (22.5)
18 months	19/19 (100)	51/52 (98.1)	70/71 (98.6)	11/19 (57.9)	23/52 (44.2)	34/71 (47.8)
24 months	18/19 (94.7)	52/52 (100)	70/71 (98.6)	12/19 (63.2)	34/52 (65.4)	46/71 (64.8)

*^a^ p = 0.02.

**Table 5:** Changes in lymphatic pathology in males and females following treatment.

Time	No. showing improvement in Lymphatic pathology/ abnormality (%)			No. showing complete recovery of abnormality (%)		
	Males	Females	Total	Males	Females	Total
6 months	38/53	13/19	51/72	1/53	2/19	3/72
	(71.7)	(68.4)	(70.8)	(1.9)	(10.5)	(4.2)
12 months	49/52	13/19	62/71	12/52	4/19	16/71
	(94.2)	(68.4)	(87.3) *^b^	(23.1)	(21.1)	(22.5)
18 months	51/52	19/19	70/71	24/52	10/19	34/71
	(98.1)	(100)	(98.6)	(46.2)	(52.6)	(47.9)
24 months	51/52	19/19	70/71	33/52	13/19	46/71
	(98.1)	(100)	(98.6)	(63.5)	(68.4)	(64.8)

*^b^ p = 0.009

There was also an improvement in the grade of lymphedema in the symptomatic subjects (6/11) as recorded by clinical examination (Fig 7A). Among the above, all three with grade I lymphedema at baseline became normal; while out of eight subjects with grade II edema at baseline, three became grade I.

Repeat ultrasonography showed disappearance of FDS in 78% of those with FDS at baseline. Two subjects remained persistently positive. Microfilarial clearance and Og4C3 antigen clearance were also noted to be greater than 90% at 18 and 24 months of follow up (Table 6).

**Table 6:** Clearance of microfilaria and adult worms following treatment.

	Individuals cleared of microfilaraemia (%) (n = 30)	Individuals cleared CFA (%) (n = 102)	FDS Clearance (n = 9) (%)
6 months	8 (26.67)	85 (83.33)	7 (77.78)
	p = 0.581		
12 months	19 (63.33)	84 (82.35)	6 (66.67)
	p = 0.478		
18 months	27 (90.0)	96 (94.11)	7 (77.78)
	p = 0.421		
24 months	28 (93.33)	97 (95.09)	7 (77.78)
	p = 0.655		

Adverse events (AEs) were seen in 9·8% (10/102) of the children following the first dose of DEC and albendazole. Following drug intake at 6, 12 and18 months the AE frequency was 8% (4/50), 2·9% (3/101) and 4% (2/50) respectively with no reports of AEs following the 24 month dose. All the adverse events were mild in nature and could be managed at home. The AEs observed included fever, headache, leg pain, nausea and light-headedness that appeared within a mean of 14 hours and disappeared within a mean of 48 hours. No serious adverse event (SAE) was reported during the study.

## Discussion

The study demonstrated a high prevalence (71·5%) of lymphatic pathology in children aged between 5 and 18 years infected with *W*. *bancrofti*. Furthermore, sub-clinical pathology was demonstrated in 63·5% of the asymptomatic but infected children. Lymphatic abnormalities were significantly more frequent (81·1%, p = 0.006) in symptomatic group subjects aged over eleven years, while there was no difference in the frequency of lymphatic abnormality (p = 0·82) in the two age groups in asymptomatic subjects. This provides the first evidence of the extent of subclinical lymphatic abnormality that develops between initial infection with *W*. *bancrofti* in early childhood and the appearance of symptomatic filarial disease in later age. The current observations in *W*. *bancrofti* infection as with *Brugia malayi* infection[10] show that sub clinical lymphatic dysfunction precedes overt disease by some considerable time. Lymphoscintigraphy is also shown to be a valuable tool for assessment of lymphatic damage in both symptomatic and asymptomatic infection in children and adults [18, 19]. It adds substantially to previously scarce demonstrations of subclinical lymphatic damage in adults demonstrated by lymphoscintigraphy, ultrasonography or nephritic manifestations [18, 20, 21, 22].

An observation of collateral lymphatic channels in lymphoscintigrams of nine (12·3%) subjects with lymphatic abnormality provides evidence for host compensatory mechanisms to prevent lymph stasis. At the same time it gives an opportunity to explore whether drug interventions are additive to the host attempts at compensating for the functional loss.

In this study, DEC and albendazole at the standard annual dose used in the Indian filariasis elimination programme progressively reduced the lymphatic abnormality or improved the lymphatic flow in 69·9% to 96·0% of subjects within 6 months to 2 years. Furthermore, the pathology/abnormality reverts to a normal picture in around two third (63%) of cases. Importantly, there was no significant additional benefit of twice annual treatment on lymphatic pathology. No gender difference was observed in the reversibility of lymphatic abnormality, although males were shown to have a higher frequency of improvement at 12 months. Similar observations were also noted for Mf positives with greater improvement at 12 months but no difference at other time points. It is clear that considerable improvement occurs within the first 6 months following initiation of treatment, but there is further progress over the next 18 months. As might be expected, there is a lag before normal function is established in around two thirds of those investigated, and it appears that some may never regain full function.

These findings are quite encouraging for the GPELF programme as it explores a new dimension of MDA against the prevailing concern that LF associated lymphedema cannot be cured [4,5]. It also substantiates the role of DEC in reversing lymphatic pathology or preventing and treating disease [23, 24, 25, 26, 27]. Studies in non-human primate models and detection of loss of motility of adult filarial worms by ultrasonography with reversal of early dilatation of lymphatic channels have already provided a suggestion that early treatment could reverse pathology [28].

These findings in *W*. *bancrofti* infection and the reversibility previously demonstrated in *B*. *malayi* infected children [10] shows the potential impact of combinations of DEC and albendazole in reversing the existing lymphatic pathology and lymphatic dysfunction [11]. Thus, it could be expected that the Global Programme will reduce or prevent overt filarial disease that can occur in 10–30% of those infected [20]. The message that many children with *W*. *bancrofti* infection already have hidden pathology, starting as early as five years of age but potentially reversible with MDA, will be useful as an advocacy tool to improve community compliance to the elimination programme in LF endemic countries, which covers an affected population of around 120 million in 83 countries, most of whom (>90%) have *W*. *bancrofti* infection [20, 28]. This should encourage funding agencies and managers of the GPELF, ultimately helping programme sustainability with the high coverage that is essential to achieve targeted elimination by 2020 [29]. It remains to be demonstrated whether similar benefits occur in *W*. *bancrofti* infections treated with ivermectin/albendazole in Africa.

LF morbidity management and disability prevention remains a critical problem in many endemic areas, despite much progress being made in interruption of LF transmission [30, 31]. Morbidity control remains a special concern since as many as 40 million have symptomatic LF disease [32]. This study has shown improvement in lymphatic abnormality as observed by lymphoscintigraphy in those with established symptoms and signs in a similar proportion (p>0·05) to those without clinical evidence of disease. Visible reduction in lymphedema was also noted in subjects presenting with early edema of the lower limb (Fig 6). The study in *B*. *malayi* infected children with LF disease also demonstrated lymphoscintigraphic as well as clinical improvement with anti-filarial drugs used in the programme [11]. This gives the hope that early treatment of lymphedema will prevent permanent disfigurement.

Observations from this study have explored an additional benefit of pharmaceutical intervention in the management of LF related disease. Thus, it can also be promoted as a tool for secondary prevention i.e., early recognition of lymphatic disease and treatment with annual DEC and albendazole.

Together with improvement or reversal of lymphatic pathology, treatment also resulted in clearance of microfilaria (93·3%) and circulating filarial antigen (95%) as well as disappearance of FDS (77·7%) in a paediatric population.

In addition to the benefits of single dose DEC and albendazole in the elimination of LF disease from endemic areas, it could also be of significance in LF non-endemic areas [33]. It is well understood that, despite threat of infection diminishing in many areas, increased travel to tropical regions exposes more people to filariasis [34]. The Geo-Sentinel Surveillance Network [35] has noted a shorter interval of presentation of *W*. *bancrofti* infection in travellers (1–6 months) after return from endemic countries. These observations highlight the need for early diagnosis and case treatment. Hence, physicians need to be aware of LF disease, its early diagnosis with clinical evaluation and lymphoscintigraphy in patients who have travelled to or are from endemic countries, while expatriates, service personnel and other travellers to endemic countries are encouraged to take personal protective measures. Equally, migrants from endemic areas need to be aware of the possibility of harbouring filariasis [35].

Thus, the present report may help to ensure treatment of early disease and prevent morbidity in those with asymptomatic pathology or early disease among non-endemic populations exposed to the risk of infection using single doses of DEC and albendazole.

## Supporting information

S1 File Protocol. (PDF)

S2 File TREND Checklist. (PDF)

S3 File Study data set. (XLSX)
