# Supplementary material for: Lymphatic pathology in asymptomatic and symptomatic children with Wuchereria bancrofti infection in children from Odisha, India and its reversal with DEC and albendazole treatment
Source: PLoS Negl Trop Dis. 2017 Oct 23;11(10):e0005631. doi: 10.1371/journal.pntd.0005631 (PMC5667936; doi:10.1371/journal.pntd.0005631)
Supplement: S1 File — (PDF) [file pntd.0005631.s001.pdf]

Am sc 24/4/09

• A study of Sub clinical lymphatic manifestation in *W.bancrofti* infection.

Protocol for a

A Multi centric study

Submitted by

Regional Medical Research Centre (ICMR), Bhubaneswar

SECTION - A

1. Names & Designations of the Investigators:

Regional Medical Research Centre (ICMR), Bhubaneswar

Principal Investigator:

Dr. S.K.Kar, M.D,
Director
Regional Medical Research Centre
Bhubaneswar, India
Tel: 0674-2301332
Fax – 0674-2301351
Email – shantanu.kar@rediffmail.com

Associate Investigators:

Dr.B. Dwibedi, M.D,
Research Officer,
Regional Medical Research Centre
Bhubaneswar, India
Email – soham2025@hotmail.com

Dr. A. Moharana, M.D,
Research Officer,
Regional Medical Research Centre
Bhubaneswar, India

Collaborator for Lymphoscintigraphy:

Prof. Dr. Birendra K. Das, M.D., FAR, ANM
Director and Chief of Nuclear Medicine
UTKAL INSTITUTE OF MEDICAL SCIENCES
357/3473 & 358/3474, Biju Pattnaik College Road
Jayadev Vihar,BHUBANESWAR - 751013

Funding:

Global Alliance to eliminate Lymphatic Filariasis
Lymphatic Filariasis Support Centre – The task force for child survival and development, 750, Commerce drive, suit 400, Decatur, Georgia 30030, Tel (404) 592-1401.

2. **Duration of the Research Project** 3 years

3. Amount of grant in aid asked for (Detailed budget in section B) - **in Indian Rupees**
Regional Medical Research Centre (RMRC), Bhubaneswar

Heads	1st Year	2nd Year	3rd Year	Total
**Staff Salaries**	10,44,400	10,44,400	10,44,400	31,33,200
**Contingency and supplies**	11,90,900	7,55,000	7,45,000	27,00,000
**Travel (TA & POL)**	3,50,000	2,50,000	2,50,000	8,50,000
**Total (in Rupees)**	25,84,400	20,49,400	20,49,400	66,83,200
**Total in US$ (@1$=Rs.49)**	52,743	41,825	41,825	1,36,393

4. **INSTITUTIONS RESPONSIBLE FOR RESEARCH PROJECT:**

Name:
Postal Address: Regional Medical Research Centre (ICMR)
Chandrasekharpur
Bhubaneswar- 751 023
Phone: *0674-2301322,2301332*
E-mail: rmrcdir@sancharnet.in Fax: +91-674-2301351

5. **Institutional Ethical clearance and Project approval:**

Enclosed

6. **DECLARATION AND ATTESTATION**

i. I/We have read the terms and conditions for ICMR Research Grant. Necessary institutional facilities will be provided if research project is approved for financial assistance.
ii. I/We agree to submit within one month from the date of termination of project, the final report and a list of articles, both expendable and non-expendable, left on the closure of the project.
iii. I/We agree to submit audited statement of account duly audited by the auditors of the Institute.

Signatures(investigators)

Dr. S.K.Kar Dr.B. Dwibedi Antaryami Maharana Dr.A.Moharana

Signatures & Seal:
Director, RMRC,
Bhubaneswar

DIRECTOR
Regional Medical Research Centre
Chandrasekharpur
Bhubaneswar-751 023

7. Is radiotagged material proposed to be used: Yes (Radio labeled material is proposed to be used for lymphoscintigraphy which is a part of medical diagnostic procedures in routine use)

8. Projects involving Recombinant DNA/Genetic Engineering work should be examined & certified by IBSC. Not applicable

9. The Institution where the study is being done should ensure that there is no financial conflict of interest by the investigators:

No conflict of interest

SECTION-B

1. TITLE OF THE RESEARCH PROJECT:

A study of Subclinical lymphatic manifestation in *W.bancrofti* infection.

2. OBJECTIVES

Objective

1. Prevalence of subclinical lymphatic pathology in population between 5-18 years with *W.bancrofti* infection in defined endemic community.

2. Effect of single annual and Biannual dose of DEC plus Albendazole on lymphatic pathology in the identified group.

3. SUMMARY OF THE PROPOSED RESEARCH WORK:

Lymphatic filariasis has been one of major public health problem in many countries globally including India. Though in India alone 31 million people are microfilaria carriers and 23 million are with clinical disease as per recent estimate. Recent reports for various region including Orissa indicate that in endemic areas around 25-30% of children of pediatric age group(0-5 years) are already infected with filarial parasite indicated by presence of circulating filarial antigen. But the course of disease development from stage of infection is not clearly delineated . The preliminary data of ongoing study at Kerala in *B. malayi* population (Shenoy et al 2007) in children between3-15years has shown, out of 100 children investigated Lymphatic abnormalities were seen in 80 cases by lymphoscintigraphy. No study has yet been addressed to find out any evidence of sub clinical lymphatic pathology in W.bancrofti infected children. As Orissa is a W.bancrofti endemic area, this study is proposed ,to study the Prevalence of clinical lymphatic pathology in population between 5-18 years with *W.bancrofti* infection and observe the effect of single annual and biannual doses of DEC plus Albendazole on the noted pathology in the identified group.

Children from Filarial endemic villages will be screened for filarial diseases and evidence for filarial infection. Before undertaking screening evaluation programme, community meetings will be arranged in the village. Information will be provided to the people on the study and its importance. Written consent will be obtained from the parents/guardian for enrolment and for subsequent investigations of the children and from subjects who are aged 18 years into the study. Clinical examination will be done and blood samples (3 ml intravenous) will be collected aseptically under supervision of clinician.

The blood samples will be brought to RMRC laboratory for Mf, CFA (Og4c3, Ag ELISA), IgG4 Ab. The children with evidence of infection or disease will be brought to the clinics at Bhubaneswar accompanied by their guardians for lymphoscintigraphy and ultrasonography and will be transported back to their villages after the investigations. The DEC-Alb intervention will be instituted in single dose as per MDA dosage supervised by the physician and the study population will be followed for a week to note any side reactions and they will be managed by research team.

Repeat evaluation by lymphoscintigraphy and ultrasound will be undertaken following drug intake for assessing the effect on lymphatic pathology and adult worm for 2 years. Blood tests and Urine examination will be repeated once yearly. For the subsequent follow up investigations also the transportation and care will be taken up by the centre.

The study is expected to provide information on magnitude of sub clinical lymphatic abnormality in the younger population in *W. bancrofti* endemic area,who are the forerunners for future development of chronic disease. Post MDA evaluation will reveal the possible

effect of DEC and albendazole on the lymphatic pathology, if substantiated, this finding will be used as an advocacy tool to boost the national MDA programme by improving community compliance.

APPLICATION OF THE WORK IN THE CONTEXT OF NATIONAL PRIORITIES OF MEDICAL RESEARCH

Global health policy targets elimination of LF with annual single dose mass drug administration for consecutive 5-6 years in the endemic countries which seems to be prolonged because of low compliance rates observed. Albendazole and diethylcarbamazine (DEC) are currently used in combination for annual mass treatment of lymphatic filariasis in all parts of the world except Africa. Lymphatic filariasis has been one of the major public health problems in many countries globally including India. An estimated 120 million people are infected globally,(Michael *et al* 1996) of which 90 % constitutes *W. bancrofti* filarial infection alone. In India alone 31 million people are microfilaria carriers and 23 million are with clinical disease as per recent estimate (Agarwal *et al* 2006). Epidemiological data indicate that the disease affects largely the poor due to unhygienic living environment where vector transmission is intense. The hall mark of the disease is episodic painful adenolymphangitis attack affecting limb or organs and irreversible grotesque. Swelling of limb or hydrocele that impairs daily activity and possess a serious social stigma. Although overt clinical manifestation of the disease have been mostly reported in young adults or higher age groups recent reports for various regions including Orissa indicate that in endemic areas around 25-30% of children of paediatric age group(0-5 years) are already infected with filarial parasite indicated by presence of circulating filarial antigen(Bal et al 2003). Earlier reports indicate detection of microfilaria infection even in infants aged 10 months. Data from endemic areas of India indicate that prevalence of filarial infection increases with advancement of age even without any overt clinical signs. Data on few asymptomatic carriers indicate morphologic distortion of lymphatic channels like dilatation and turtuosity that are ascribed to the sub clinical pathology caused by adult parasite. Although large group of paediatric population are infected the clinical disease is clustered in later age groups. The preliminary data of ongoing study at Kerala in *B. malayi* population (Shenoy *et al* 2007) indicated the presence of live adult worms in 14% of children between age of 3-15 years(six microfilarimic children and eight amicrofilarimic children with history of clinical diseases had adult adult worm). Out of 100 children investigated lymphatic abnormalities was seen in 80 cases by lymphoscintigrapy(Shenoy *et al* 2007). No study has yet been addressed to find out any evidence of subclinical lymphatic pathology in W.bancrofti infected children and adults till clinical signs appear and there after.

Currently as per the global programme for elimination of lymphatic filariasis (Ottensen E A 1998) most endemic countries including India have initiated annual single dose mass administration of DEC for 5 to 6 yrs consecutively with a target towards elimination. Where MDA is targeted to interrupt transmission, morbidity management tool is being utilized to address the overt cases with chronic clinical manifestations.

However the role of MDA in already infected children who have not developed symptoms is not addressed in MDA due to paucity of any evidence. Even effect of MDA on clinical filariasis is yet to be evaluated.

The study will give information on magnitude of sub clinical lymphatic abnormality in the younger population in a *W. bancrofti* endemic area, who are the forerunners for future development of chronic lymphatic disease. Post MDA evaluation will reveal the possible effect of DEC and Albendazole on the lymphatic pathology. If substantiated, this finding will boost the national MDA programme by improving community compliance. Reversibility of clinical manifestation if found will be a new addition to MDA advocacy tools. This study would also add to the primary prevention targeted by the MDA programme through

prevention of disease transmission by supplementing evidence of secondary prevention by early treatment.

4. PRESENT KNOWLEDGE & RELEVANT BIBLIOGRAPHY

Lymphatic filariasis has been one of the major public health problems in many countries globally including India. An estimated 120 million people are infected globally,(Michael *et al* 1996) of which 90 % constituted by *W. bancrofti* filarial infection. In India alone 31 million people are microfilaria carriers and 23 million are with clinical disease as per recent estimate (Agarwal *et al* 2006). Epidemiological data indicate that the disease affects largely the poor due to unhygienic living environment where vector transmission is intense. The hall mark of the disease is episodic painful Dermato-lymphangio-adenitis (ADLA) attack affecting limbs or genital organs that result in irreversible, grotesque deformities. Swelling of limbs and hydrocele impair daily activity causing serious social stigma. Although overt clinical manifestations of the disease have been mostly reported in young adults or older age groups, recent reports from various regions including Orissa indicate that in endemic areas around 25-30% of children (0-5 years) are already infected with filarial parasite as indicated by presence of circulating filarial antigen (Bal et al 2003). Earlier reports indicate detection of microfilariae (mf) even in infants aged 10 months. Data from endemic areas of India indicate that prevalence of filarial infection increases with advancement of age even without any overt clinical signs. Data on few asymptomatic carriers indicate morphologic distortion of lymphatic channels like dilatation and tortuosity, that are ascribed to the sub clinical pathology caused by the adult parasite. Although large group of paediatric population is infected, the clinical disease is clustered in later age groups. The preliminary data of ongoing study at Kerala in *B. malayi* infected population (Shenoy *et al* 2007) indicated the presence of live adult worms in 14% of children between age of 3-15 years (six microfilarimic children, one with filarial disease and 7 who were only positive for the filariasis specific IgG4 antibody test). Out of 100 children investigated lymphatic abnormalities was seen in 80 cases by lymphoscintigraphy (Shenoy *et al* 2007). No study has yet been done, that addressed to find out any evidence of subclinical lymphatic pathology in *W.bancrofti* infected children and adults before the appearance of clinical signs and there after.

Currently as per the global programme for elimination of lymphatic filariasis (Ottensen 1998) most endemic countries including India have initiated annual single dose mass administration (MDA) of DEC for 5 to 6 yrs consecutively with a target towards elimination. While MDA is targeted to interrupt transmission, morbidity management tool is being utilised to address the overt cases with chronic clinical manifestations.
However the role of MDA in already infected children who have not developed symptoms is not addressed in MDA due to paucity of any evidence. Even effect of MDA on clinical filariasis is yet to be evaluated.

REFERENCES:

1. Agarwal, V.K (2006) Lymphatic filariasis in India, MJAFI (EXPAND THIS), 62:359-362.
2. Michel A , Bundy DAP and Grenfelt BT (1996). Re-assessing the global prevalence and distribution of lymphatic filariasis. Parasitology. 112, 409-428.
3. Shenoy, R.K *et al* (2007). Preliminary findings from a cross sectional study on lymphatic filariasis in children in an area of India, endemic for Brugia malayi infection. Annals. Trop.Med.Parasitol. Apr: 101(3): 205-213.

4. Shenoy, R.K et al (2007). Doppler ultrasonography reveals adult worm nests in the lymph vessels with brugian filariasis. Annals. Trop.Med.Parasitol. Mar: 101(2): 173-180.
5. Ottensen E A (1998) In infectious Diseases and Public Health(ed. Angelico M and Rocchi G) Balaban Publishers, Tel Aviv pp : 58-64.
6. Bal M.S and Das, M.K. (1999) Antigenicity of a filarial protease from Setaria digitata in W.Bancrofti infection.Annals of Tropical Medicine and Parasitology 93: 279-288.
7. Bal M.S, M.K.Beuria, N.N.Mandal and M.K.Das (2003) Parasite antigenaemia and IgG4 antibodies to a filarial protease in an endemic human population in India. Journal of Helminthology 77: 287-290.

5. PRELIMINARY WORK ALREADY DONE BY THE INVESTIGATORS:
The centre is engaged on research on priority areas of filariasis for last 25 years. The investigators have conducted several open level and blind trials on efficacy and safety of the antifilarials like DEC and Ivermectin at different dosage levels. Results of these activities have been reported and published with lots of implications to the global programme for elimination of LF.
Presently the investigator are also continuing research on efficacy of lower dose of DEC in MDA mode in endemic villages and also conducting an open level clinical trial on dose and interval modification of DEC and Albendazole on microfilaria clearance.

The centre has already established several field areas nearby, where filariasis is endemic; suitable for both long term & short term studies. RMRC runs a out-patients clinic at Capital Hospital, Bhubaneswar with facilities for lab diagnosis & treatment including morbidity management facility like decompression therapy. This centre has linkages with the Medical Colleges of the state in relation to the patient database, OPD & in-patient facility & scientific interactions in relevant projects.

In context to the proposed project the centre has identified the endemic villages for selection of the subjects for the study and the filarial disease spectrum in the population has been evaluated as a part of the field survey necessary for the project.

6. LINKS WITH OTHER PROJECTS: The completed and ongoing projects on efficacy, tolerability and dose ranging of antifilarials are all related and in line with the global and national programme for elimination of LF and another on going project is effect of Albendazole dose and interval on *Wuchereria Bancrofti/Brugia malayi* microfilarial clearance in India, a randomized, open label study. The proposed study on lymphatic pathology will be additive to the above international relevant to the national MDA programme or LF elimination.

7. LIST OF IMPORTANT PUBLICATIONS/ NATIONAL and INTERNATIONAL PROCEEDINGS:

1. Lymphatic nodules in human filariasis. **S.K.Kar**, Lymphology, 18, 4(1985), 163-164.
2. Tissue Tonometry: A useful tool for assessing Filariasis Lymphedema. **S.K.Kar,** P.K.Mania, Jour. Of Lymphology, 25 (1991) 51-61.
3. Ivermectin in the treatment of Bancroftian Filaria infection in Orissa (India), **S.K.Kar**, Patnaik, S., J.Mania, V.Kumaraswamy; South-east Asian Journal of Trop. Medicine and public health, 24(1), (1993), 80-86.
4. Humoral Immune response in filarial fever in bancroftian filariasis, **S.K.Kar**, J.Mania, P.K.Kar. The Trans. Of Roy. Soc. Of Tropical Med. & Hyg. 87 (1993), 230-233.
5. The sheath of Mf of *Wuchereria bancrofti* has Albumin and Immunoglobulin on its surface. **S.K.Kar**, J.Mania, C.Baldwin, D.A.Denham. Parasite Immunology, 15 (1992), 297-300.
6. Side reactions following Ivermectin Therapy in high density Bancroftian Microfilaraemics. **S.K.Kar**, S.Patnaik, V.Kumaraswamy, R.S.N.Murty. Acta Tropica, 55 (1993), 21-31.
7. Prevalence of Lymphatic nodules in bancroftian endemic population. **S.K.Kar,** J.Mania, P.K.Kar. Acta Tropica, 55 (1993), 53-60.

8. Clinical filarial disease in two ethnic endemic communities of Orissa. **S.K.Kar,** J.Mania, P.K.Kar, Jour. Of Trop. Med. & Hyg. 96 (1993), 311-316.

9. Atypical features in Lymphatic Filariasis. **S.K.Kar**. Ind. Jour. Of Med. Res. 84 (1986), 270-274.

10. Epidemiological study on Lymphatic Filariasis in an endemic zone of Orissa. **S.K.Kar**. Jour. Of Communicable Disease, 18 (1986), 312.

11. Tropical pulmonary eosinophilia in an Orissa children. **S.K.Kar**, J. Mania. The National Medical Journal of India, 6(2), 1993, 63-64.

12. The use of Ivermectin against scabies. **S.K.Kar**, J.Mania, S.Patnaik. The Nat. Medical Jour. Of India, (1994).

13. Detection of albumin and immunoglobulin on surface of *B.malayi* mf immunoflurescence. J.Mania and **S.K.Kar.** The Indian Journal of Medical Research (1994).

14. The cross-sectional epidemiological studies on Kala-azar around Patna city in Bihar. D.S.Dinesh, R.C.Dhiman, A.Ranjan, S.K.Prasad, K.Kishore and **S.K.Kar.** Indian Journal of Parasitol. 18 (1994) pp. 143-147.

15. Babu BV and **Kar SK** (2004) Coverage, compliance and some operational issues of mass drug administration during the programme to eliminate lymphatic filariasis in Orissa, India, *Tropical Medicine and International Health* (In Press).

16. Randomized clinical trial to compare the efficacy of three treatment regimus along with food care in the morbidity management of filarial lymhedema. A. S. Kerketta, B. V. Babu et al. Tropical Medicine and International health, 10(7) 698-705.

17. Clinician's practice related to management of filarial adenolymphongitis and lymphodema in Orissa, India, A.S. Kerketta, B. V. Babu, B. K. Swain Acta Tropica- 102 (2007) 159-164.

18. A successful outcome of gross haematuria treated with DEC and Ivermectin , Kerketta, A. S., K. Dhal, R.N. Nayak (in Press) Trans of Royal Soc. Of Trop Med and Hyiegene.

19. Assessment of Therapeutic efficacy of chloroquine in the treatment of uncomplicated falciparum malaria in a tribal block of Kalahandi district of Orissa, India. Kerketta, A. S. S. S. Mohapatra, Kar, S. K, Tropical Doctor (in Press)

20. .Pathology and pathogenesis of lymphatic filariasis. Proceedings of symposium on filariasis; S.K.Kar, XXXVII Annual Conference of Indian Association of Pathology and Microbiologist, Calcutta, 18th Dec. 1988.

8. DETAILED RESEARCH PLAN

8.1 STUDY DESIGN AND METHODS

STUDY DESIGN

Selection of *W.bancrofti* endemic village with mf rate 5% or more
and population around 6000
(5-18 years –2500)

↓

Screening - clinical examination, Mf, CFA and IgG4,

Proportion of prepubertal age(5-11) around 30 and pubertal(12-18) around 20

Children with evidence of infection (Mf + /,CFA+)

(n=50)

Clinical filariasis (Lymphedema, ADLA or hydrocele) with either CFA/IgG4/mf +ves/Haematuria

(n=50)

↓

Lymphoscintigraphy of upper and lower limbs and Doppler ultrasonogram for adult worm in scrotum, breasts and other preferred sites in 4 limbs.
(n=100)

↓

Abnormal scan or FDS positive in any site

↓

Intervention with DEC and alb (single dose supervised by research team, side reaction monitoring and management if any)
(Randomised to two groups,
Age group and category represented)

Annual dose (n=50) | Biannual dose (n=50)

↓

Repeat all tests - at 6, 12 ,18 and 24 months

↓

Result analysis and interpretation

Study Area and population

Based on the findings of ongoing study of the centre, mf prevalence rate of endemic communities varies from 5-8% and an estimated 40% of population (Dejure) are within the age range of 5-18 years. Hence endemic villages of Khurda and Cuttack with mf prevalence rate of more than 5% will be selected as study area. Circulating filarial antigen positivity is also reported to be around 18 to 30% in the endemic villages of Orissa that have been studied. Villages of around 6000 population in total will be selected from the endemic area around Bhubaneswar in Khurda and Cuttack district for the study. The population between 5-18 years (approximately 2500 (i.e 40% of the total population) will be screened for clinical filarial disease, mf, CFA and antibody (IgG4) to *W.bancrofti* infection. This is expected to provide around 200 children with evidence of filarial infection (mf or CFA or IgG4 positivity). These children will be classified into two following groups; i) Asymptomatic with evidence of infection (mf and/or CFA positives) ii) Clinical filarial disease with microfilarimia, antigenimia or antibody positivity to W.b. In total, 100 children will be selected for the study so that each study group will have 50 individuals. All these population will be subjected to initial clinical examination, urine examination, mf, CFA, IgG4 tests, Doppler ultrasonogram (for adult worms) and lymphoscintigraphy of the limbs (for lymphatic pathology).

Rationale for the age group and the categorisation:

A) Age Groups: (5-18 years)

While reversibility of subclinical pathology has been shown in *B.malayi* positive children in prepubertal age group i.e. 3-15 years, the post pubertal group (12-18 yrs) which has not been studied so far, is included in this study. The importance of post pubertal group lies in the opportunity to study the possible effect of the treatment in the reversal of the overt clinical manifestation like lymphedema, ADLA and hydrocele. The age group of 3-4 years with evidence of filarial infection although will be available, keeping in mind the practicability and feasibility of subjecting such young group to lymphoscintigraphy, it may be little difficult to include this group in the study; unlike in the *B.malayi* study by Dr.Shenoy and his team in Kerala where literacy rate is high and the study areas are better developed.

B) Classification of Groups/Categories:

The aim of the project is to identify those with subclinical or early pathology and those with overt disease to see their response to MDA. Hence effort is taken to include all category of cases presenting in different spectrum of filarial infection. The treatment effect will be later assessed in each category represented adequately by their age, for lymphatic change (Prepubertal, post pubertal children and adults)

Catagories:

1. Asymptomatic with evidence of infection (mf &/or CFA positives) ***(n=50)***

Since mf prevalence in endemic communities is around 5%, screening of study population will yield required number of cases in all age groups (prepubertal 5-11 years, n= 30, post pubertal children 12-18 years, n= 20) to include proportionately in study.

2. Clinical disease:

It is important to know the effect of the drug on clinically discernible cases of filarial disease for its reversibility, both by reversibility of lymphatic abnormality and/or disappearance of overt clinical signs. Hence cases will be recruited from post pubertal age groups as well as from pre pubertal age(with any evidence of filarial infection) presenting with lymphoedema, ADLA and/or hydrocele. Since haematuria has been reported to be one manifestation of the

disease, though not commonly encountered in these endemic areas, efforts will be taken to include such cases also if available.

Methodology

After selection of the village the population census of the target age group will be made. Written consent will be obtained from the parents / guardian for enrolment of the children and from adults aged 18 years into the study and for subsequent investigations as per protocol. Clinical examination will be done and blood samples (3 ml intravenous) will be collected aseptically for screening of mf (1ml by nucleopore membrane filtration), CFA(Og4C3 Ag ELISA) and IgG4(*Wb* specific by ELISA) antibody. Efforts will be taken to also include cases of haematuria, by routine examination of urine in all subjects. These tests will be carried out at RMRC laboratory following standard procedures. The children with evidence of infection or disease, accompanied by their guardians will be brought to the clinics at Bhubaneswar for lymphoscintigraphy and Doppler ultrasonography and will be transported back to their villages after the investigations. For the subsequent follow up investigations also the transportation and care of the subjects will be undertaken by the centre. The DEC-alb intervention will be instituted in single dose (standard drugs procured by centre from reputed firms) supervised by the physician and the study population will be followed for a week to note any side reactions and they will be managed by research team.

All study population will be enrolled into 2 groups in equal numbers (50 each) representing each subgroup, taking care of the two age groups i.e.5-11 and 12-18. Subjects enrolled in the study would not receive MDA or any antifilarials from other sources.during the study period

Drug doses(As per MDA dosage)
5-14 yrs- DEC 200mg + ALB 400 mg
15-18 yrs- DEC 300 mg + ALB 400mg

Laboratory Methodology:

Mf count, CFA, IgG4 Test and urine examination

Mf count will be done from 1 ml of intravenous blood by membrane filtration and standard staining procedure.

Quantitative ELISA will be used for measurement of circulating filarial antigen for detection of Og4C3 and IgG4 (Bal *et al* 1999) tests will be done by using standard immunological procedures using Wb specific antigen. Urine examination for macro or microscopic haematuria will e done in all enrolled subjects.

Ultrasonography:

Ultrasonography will be carried out by skilled ultrasonologist at Bhubaneswar using 8-11MHz transducer colour Doppler scan. In the lower limbs both inguinal regions, medial aspects of thighs and popliteal region ; in the upper limbs both axillary regions and inner aspects of upper arms up to epitrochlear region, scrotal sacs in post pubertal males (including hydroceles) and breasts in post pubertal females will be scanned for detection of live adult worm nests and FDS. Procedure will be followed as per the referred study at Kerala.

Lymphoscintigraphy:

Lymphoscintigraphy of both upper and lower limbs will be carried out at Bhubaneswar by a nuclear medicine specialist using radio labelled "Tc^{99m} tagged sulphur colloid (BARC, Mumbai, India)" . The procedure will be standardised using similar methodology carried out in Dr.Shenoys' study to maintain uniformity for interpretation. The children undergoing the procedure will be observed for 6 hours before being discharged.

Inclusion criteria:.

1. Written consent from parents or guardians(for children below 18 years) and from those aged 18 years.
2. Children between 5-18 years age
3. Evidence of filarial diseases like ADL, lymphedema and hydrocele.
4. History of filarial disease or past microfilaraemia (Since they are known to have subclinical damage to lymphatics caused by the adult worms).
5. Evidence of present infection i.e. mf positive or CFA positive or antibody positive for *W.bancrofti*.

Exclusion criteria:

1. Any systemic illness including cardio vascular or respiratory disease.
2. Abnormal liver and kidney function tests
3. Peripheral vascular diseases.
4. History of drug allergy to DEC/albendazole.
5. H/o taking antifilarials in the last one year

Intervention and Assessment:

The study population (5-18 yrs) will receive DEC plus albendazole in MDA doses. Drug allocation into two groups i.e **Annual and Biannual,** will be done by statistician keeping the assessee blinded. Care will be taken to include both age groups in all categories proportionately. Both drugs, DEC and alb will be procured from standard reputed firms by the centre and the single (annual/biannual) dose will be given under supervision of the team physician. The population will be kept under supervision for a week for observation and management of any side reactions. Repeat evaluation by Lymphoscintigraphy and ultrasound will be done 6 monthly following drug intake for assessing the effect on lymphatic pathology and adult worm for 2 years. Blood tests and urine examination will be repeated once yearly.

8.2 Responsibility of Investigators/ Collaborators

RMRC investigators- The investigators(PI and Associates) from RMRC, Bhubaneswar will carry out and be responsible for all the project activities like village selection, subject enrollment , the laboratory procedures, Intervention and follow up activities mentioned above along with data analysis and reporting. Because certain special facilities/ expertise are not available at this centre, the investigators will take assistance from an Ultrasonologist, experienced in the field and Dr. B.K.Das, Nuclear Medicine Expert for the procedures: Ultrasonography detection of adult worm and Lymphoscintigraphy respectively. How ever the RMRC investigators will be responsible for the final interpretation of the tests carried out with inputs from the above experts.

Collaborator (Lymphoscintigraphy) Prof. Dr.B.K.Das:– will provide the facility and undertake Lymphoscintigraphy of the study subjects and provide his comments on the test results. He will be assisting in interpretation of the Lymphoscintigraphy results. The subject selection and transport to the Lymphoscintigraphy center will be done by the RMRC team.

8.3 DATA ENTRY AND ANALYSIS

The collected information will be entered into SPSS programme and will be analysed after data verification.

8.4. Description of Consent Procedures

Informed consent is a process that is initiated prior to the individual's agreeing to participate in the study and continuing throughout the individual's study participation. Consent forms describing in detail the study agent/intervention(s), study procedures and risks are given to the subject and written

documentation of informed consent is required prior to starting study agent/intervention. Consent forms will be IRB-approved by the RMRC, Bhubaneswar and the subject will be asked to read and review the document. Upon reviewing the document, the investigator will explain the research study to the subject and answer any questions that may arise. The subjects will sign the informed consent document prior to any procedures being done specifically for the study. The subjects may withdraw consent at any time throughout the course of the trial. The rights and welfare of the subjects will be protected by emphasizing to them that the quality of their medical care will not be adversely affected if they decline to participate in this study. All subjects will have the goals, procedures and risks of the study explained in the local language or English and will be asked to sign a consent form. The administrator of the consent form will sign and date the form. The protocol and the consent to be given to each volunteer will be approved by the ethical committees of the centre.

In the situation whereby the subject cannot read or write, the study team will go over the consent document verbally with the subject, make certain that he/she understands the consent, after which a thumbprint will be used instead of a signature.

8.5. Plan for Maintaining the Privacy and Confidentiality of Subject Records

All records will be maintained in a locked file at the study sites where they are accessible only to authorized personnel.

8.6 DATA AND SAFETY MONITORING PLAN

1. Data Monitoring Plan

Source documents are original documents, data, and records from which the subject's data are obtained. All essential documentation for all study subjects including history and physical findings, laboratory data, and results of consultations will be maintained by the investigators in a secure storage facility for a minimum of three years. These records are to be maintained in compliance with IRB/EC, state and federal requirements, whichever is longest. All records are to be kept confidential to the extent provided by federal, state and local law. It is the investigator's responsibility to retain copies of source documents until notified. Should the investigator wish to assign the study records to another party or move them to another location, they must notify in writing of the new responsible person and/or the new location.

Data collected on case report forms will be keyed into a database by data entry personnel that are part of the automated data processing group and copies of electronic data will be retained in a password-protected fashion.

2. Data management plan

Data will be collected on the Case Report Forms (CRFs), which are the source document. Data will be double entered into RMRC, Bhubaneswar, database for analysis. Data from the project will be monitored by the P.I. and co-investigators every month with assistance from the statisticians as needed. Regulatory agencies will also have access to the data.

8.7 REMUNERATION PLAN FOR SUBJECTS

Transportation costs and care will be provided for each study subject as will be compensation for wages/work /activity lost for the study subject for the visit days. Although there will be no limit on travel/wage/activity loss reimbursement, it is expected that it will not exceed Rs 200.00 per day. All participants will be given a screening identification card.

REGIONAL MEDICAL RESEARCH CENTRE, BHUBANESWAR

BUDGET

Budget requests (in Indian Rupees)					
**1. Personnel**		**Salary/ Month**	**Year 1**	**Year 2**	**Year 3**
**Principal Investigator:** Dr.S.K.Kar, Director			Need no honorarium for the project		
**Associate Investigators**					
Dr.B.Dwibedi<br>Dr.A.Maharana			Need no honorarium for the project		
**Temporary project staff**					
1.Medical Officers(Two)<br>2.Research Associate(One)<br>3.Laboratory Technician (Two)		25000<br>22000<br>15000	6,00,000<br>2,64,400<br>1,80,000	6,00,000<br>2,64,400<br>1,80,000	6,00,000<br>2,64,400<br>1,80,000
**Total personnel Yr Wise**			**10,44,400**	**10,44,400**	**10,44,400**
**Total personnel for 3 years**					**31,33,200**
**2. Contingency and supplies**					
**A. Field Screening camp costs** (Meeting arrangement, Audio visual Show etc.	(Rs 5000 X 10 camps)		50,000	nil	nil
**B. Chemicals & Kits**<br>Og4C3, Nuclepore filter ,Reagents for haematological, biochemical tests	Screening by lab tools for identifying eligible at initial phase and tests for 6 monthly follow up (5no.)		2,50,000	50,000	50,000
**C. Supplies and Miscellaneous Expenses**<br>Glass/plastic ware<br>Field and Office stationery, common medicine for primary health care and trial drug (DEC+ Albendazole), communication and postage etc.			3,30,000	2,25 ,000	2,25,000
**D. Patient cost**<br>Wage compensation and patient care for individuals enrolled.			75,000	60,000	60,000
**E. Investigating charges**					
Lymphoscintigraphy and Ultra sound charges			4,85,000	4,20,000	4,20,000
			**11,90,000**	**7,55,000**	**7,45,000**
**Total patient cost for 3 years**					**27,00,000**
**3. Travels**					
Dearness allowance of staffs for travel to field areas.			1,50,000	100000	100000
POL & Vehicle Maintenance for travel to field and transportation of children to investigation centre.			2,00,000	1,50,000	1,50,000
**Total Travel Yr Wise**			**3,50,000**	**25,0,000**	**2,50,000**
**Total Travel for 3 years**					**8,50,000**

[illegible]nd Total year wise in Rs	25,84,400	20,49,400	20,49,400
Grand Total in US$ (Approx @1$=Rs.49)	52,743	41,825	41,825
Grand Total for 3 years in Rs			66,83,200
Grand Total for 3 years in US$ (Approx @1$=Rs.49)	1,36,393		

Budget Justification

1. Personnel:

Medical officers (Two)-Project activities require extensive field screening of population aged 5-18 years for identifying the eligibility of subjects by clinical examination and laboratory diagnosis for infection. Besides the children are to be brought to the investigation centre for Lymphoscintography and ultrasound. Also the children will be followed up six monthly. All the above activities need dedicated medical officers skilled in clinical examination and supervision of field activities .He/she will support the nuclear medicine expert during the procedure of investigation for cooperation of children in the investigation process.
The medical officers will be involved in the prescreening field activities which will include village meetings, motivation and counseling activities involving parents as well as studied children and the consent process. He will organize screening camps and monitor the field activities. They will conduct clinical examination and supervise sample collection. They will also provide primary health care to the study subjects which is quite essential to get cooperation for the study and it is a part of ethical responsibility to take care of the children under the study. The children will be transported to the investigation clinic under their care. During the procedures like Lymphoscintography and ultrasonography, the MOs will also take care of preparedness of the children for the investigations. They will also follow the children in the field after supervised intake of DEC+Albendazole for monitoring and management.

Research Associates (One)-
The proposed research associates will monitor sample storage in the laboratory, conduct serological test (Og4C3 and IgG4) and monitor all other laboratory test. He/she will maintain the laboratory records and enter the data in computer. Also assist in data management analysis, interpretation of data, preparation of reports related to the study.

Lab technicians (Two) –The project involves population motivation, screening of children, collection of intravenous blood sample, transport of samples and transportation of study children for investigations. During screening, around three thousand children will be included for collection of intravenous blood samples. So, the lab technicians to be recruited would be expert in drawing of blood from children. The lab technicians will be engaged primarily for field activities along with routine procedures invoved.The proposed lab technician will be engaged in the following activities - 1.Assisting the medical officer in field camps, motivation and consent process, 2. Sample collection (IV blood), coding of samples, sample transport & preservation, 3.Laboratory tests (Mf count, hematological and biochemical test) & lab maintenance, and 4. Transport and care of children to investigating clinic and back

2. Contigency and supplies:

Chemicals ,kits and supplies are required for collection of about 3000 blood specimens during initial screening from the endemic population (keeping Mf positivity rate of 4% in the endemic community), separation of sera, aliquoting of samples, transportation to main laboratory and storage. Micropipettes, chemicals and plastic and glassware are required for conducting the ELISA tests, biochemical tests & hematological counts which will be conducted for the initial screening and subsequent 6 monthly follow-up.

A. Field Screening camps-
Field camp will be organized for population motivation and identify eligibles for the study.To get cooperation for blood collection and participation in the study, local persons will play a vital role. To

get them into confidence, community awareness and health education programmes as well as mass health camps will be a prerequisite. For this public meetings, group discussion & audio visual demonstration etc will be arranged with help of panchayat, village heads & health personnel of the locality. For these activities, leaflets, booklets, pamphlets and posters will be prepared. Banners and Audio visual aids will be used to explain and educate the public. Approximately Rs 5000/- will be utiliized towards meeting arrangements, mike announcements, audio-visual hiring etc. and ten such camps are planned(total Rs 50,000/-) in the first year.

B. Chemicals and kits-

Relevant chemicals and kita will be procured which includes staining materials, nucleopore filters, ELISA kits for Og4C3 and IgG4, hematological and biochemical tests like DC/TLC, Hb%,urea and creatinine for analyzer equipment.

C. Supplies and miscellaneous expenses-

Disposable syringes, needles, gloves, vacutainers, collection and storage vials, microtips, glass slides etc. will be procured. Formats of clinical examination, personal data sheets, and consent forms will be printed. Stationary items will require for storage, labeling, transport of specimens. Stickers, gums, packaging items, (cool packs / dry ice as per availability and requirement) will be purchased. Computer stationary like CDs/ floppy, printing accessories will be used for data entry and reporting. Medicines will be utilized for primary treatment of the examined subjects. DEC, Albendazole and additional drugs for side reaction management will be procured for the enrolled subjects.

D.Patient Costs-

It will cover the cost towards wage compensation (Rs 200/day), and other expenses towards patient care (food etc.) for the enrolled children and their accompanying guardians to the investigation clinic.

E. Investigation charges-

It will cover the cost to be paid for Lymphoscintography and ultra sound examination of the eligible children at base line and subsequent follow up 6 monthly for three years.

3. Travel & Allowances:

Study site is approximately 70 kms from this centre. Frequent visits will be required to the field site for continuous activities like community awareness, sensitization & motivation, screening of the individuals, transportation of the patients to & from the investigation clinic in Bhubaneswar & follow-up of the treated individuals. POL & vehicle maintenance will be required for the above visits. Fuel will be required at the approximate mileage of 9-10 kms using RMRC vehicle.

Dearness allowances will be given to the staffs (six) attending the field at the admissible rate under ICMR guidelines.
